# A calibrated agent-based computer model of stochastic cell dynamics in normal human colon crypts useful for in silico experiments

**DOI:** 10.1186/1742-4682-10-66

**Published:** 2013-11-18

**Authors:** Rafael Bravo, David E Axelrod

**Affiliations:** 1 Department of Genetics, Rutgers University, 604 Allison Rd, Piscataway, NJ 08854-8082, USA; 2 Department of Computer Science, Rutgers University, 110 Frelinghuysen Rd, Piscataway, NJ 08854-8019, USA; 3 Rutgers Cancer Institute of New Jersey, 195 Little Albany Street, New Brunswick, NJ 08901-1998, USA

**Keywords:** Agent-based model, Colon crypts, Cell dynamics, Stem cells, Adenomas, Colon cancer, Chemotherapy, Radiation therapy

## Abstract

**Background:**

Normal colon crypts consist of stem cells, proliferating cells, and differentiated cells. Abnormal rates of proliferation and differentiation can initiate colon cancer. We have measured the variation in the number of each of these cell types in multiple crypts in normal human biopsy specimens. This has provided the opportunity to produce a calibrated computational model that simulates cell dynamics in normal human crypts, and by changing model parameter values, to simulate the initiation and treatment of colon cancer.

**Results:**

An agent-based model of stochastic cell dynamics in human colon crypts was developed in the multi-platform open-source application NetLogo. It was assumed that each cell’s probability of proliferation and probability of death is determined by its position in two gradients along the crypt axis, a divide gradient and in a die gradient. A cell’s type is not intrinsic, but rather is determined by its position in the divide gradient. Cell types are dynamic, plastic, and inter-convertible. Parameter values were determined for the shape of each of the gradients, and for a cell’s response to the gradients. This was done by parameter sweeps that indicated the values that reproduced the measured number and variation of each cell type, and produced quasi-stationary stochastic dynamics. The behavior of the model was verified by its ability to reproduce the experimentally observed monocolonal conversion by neutral drift, the formation of adenomas resulting from mutations either at the top or bottom of the crypt, and by the robust ability of crypts to recover from perturbation by cytotoxic agents. One use of the virtual crypt model was demonstrated by evaluating different cancer chemotherapy and radiation scheduling protocols.

**Conclusions:**

A virtual crypt has been developed that simulates the quasi-stationary stochastic cell dynamics of normal human colon crypts. It is unique in that it has been calibrated with measurements of human biopsy specimens, and it can simulate the variation of cell types in addition to the average number of each cell type. The utility of the model was demonstrated with *in silico* experiments that evaluated cancer therapy protocols. The model is available for others to conduct additional experiments.

## Background

Colon crypts are a paradigm of a dynamic biological system that maintains homeostasis. Crypts contain several types of cells with transitions between cell types, and variation in the number of each cell type. The transitions between cell types and the variation in the number of each type of cell cannot be observed *in vivo* in real time, but have been inferred from static histological images. Computer and mathematical models based on information obtained from these static images and from molecular cell biology experiments have provided insights into these dynamic processes.

### Biology of crypts

Crypts are invaginations of the lumen of the large intestine (colon) and of the small intestine. The crypts of the colon function to absorb water and exchange electrolytes from the feces, and to produce mucus to lubricate feces as they move through the colon [1]. Each human crypt contains several thousand cells arranged in the form of a test-tube open to the lumen of the colon. Stem cells near the bottom of the crypt may be quiescent or may become active and divide to produce proliferating cells [2]. As the proliferating cells move up the crypt they have a reduced probability of dividing and an increased probability of differentiating [3]. In the normal colon the production of new cells is balanced by the loss of old cells. This balance is altered in colon cancer.

Most of what we know about crypts has been obtained by experiments with mouse tissue, rather than human tissue. This is because mouse tissue is more readily available than human tissue, and mice can be genetically altered. Genetically engineered mice have been a powerful tool to reveal the function of many molecules controlling intestinal crypt cell proliferation, differentiation, and lineage [4]. The intestinal crypts of mice differ from human colon crypts in several ways. Mouse crypts are smaller than human crypts. Intestinal crypts are associated with villi, but colon crypts do not have villi. Intestinal crypts have some cell types that are missing or not readily recognizable in colon crypts. We have been able to obtain normal human colon biopsy tissue, and based on measurements of the number of each cell type in multiple crypts, have for the first time developed a calibrated model of cell dynamics in normal human colon crypts.

### Cell dynamics

Mathematical and computer models can incorporate features that have been observed in static stained tissues, and by simulations, can provide insights about cell dynamics that are not directly available from static images. The dynamics of cell proliferation in crypts have been described by deterministic models or by stochastic models. Some models have made use of estimates of the average number of each cell type in a typical crypt. Deterministic models have described the numbers of each cell type, the transition rates between cell types, the influence of one cell type on the proliferation of other cells types, and the stability of the total number of cells per crypt [5,6]. However, we have observed a variation in the number of each cell type in adjacent crypts in biopsies of normal colon tissue. This variation indicates that the average number of each cell type is not sufficient to characterize cell dynamics. This suggested to us, and others [7,8] that the variation in numbers of cells over time, around an average, can be accounted for by a model that incorporates a stochastic quasi-stationary process [9].

### Plasticity of cell types

In some previous biological descriptions of colon crypts [10,11], and in some previous mathematical models of colon crypts [5,6] each cell is considered to have an intrinsic type, either stem cell, proliferating cell or differentiated cell.

Stem cells are thought to be in a specialized region, the stem cell niche, in which they respond to signals that control their maintenance and probability of division [12-19]. The molecules Dickkopf-1 (Dkk1) and the secreted frizzle related protein 1 (sFRP1) are thought to keep quiescent stem cells in the niche from dividing [20,21]. However, quiescent stem cells may transition to active stem cells [2]. In order to take this transition into account we have modeled the transition from quiescent stem cells to active stem cells as the result of stem cells leaving the niche.

There is recent experimental evidence that cell types are not fixed but can interconvert [21-26]. This has led to the concept of dynamic plasticity of cell types [27-30]. Several published models have incorporated the concept of plasticity, in which cell types are dynamic and may interconvert [8,31,32]. Other mathematical models have described transitions between multiple compartments of one cell type [33,34]. Many older mathematical models of crypt dynamics have been discussed in a comprehensive review [35].

The inter-conversion of cell types may be influenced by adjacent cells and by extracellular molecules, singly or in combination [36]. Measurements by gene expression arrays and *in situ* hybridization indicate that there are gradients of molecules along the crypt axis [18,37-40]. Many of these molecules have been associated with the functions of cell proliferation and cell differentiation. Some, such as Wnt have a positive effect [41], and others such as Dkk1 [42] have a negative effect. The model presented here assumes that the inter-conversion of cell types can occur as a cell moves in molecular gradients along the crypt axis.

### Monoclonal conversion

Monoclonal conversion refers to a crypt in which all cells are derived from a single progenitor cell [3]. Monoclonal conversion could be thought of as occurring by selection or by a random process. In the former process, only one of many possible quiescent progenitors more efficiently begins to proliferate, or produces more rapidly dividing progeny. In the latter process, many progenitors produce progeny clones, then without selection there is extinction of most clones, and stochastic survival of only one clone. The latter is referred to in population genetics as neutral drift.

Monoclonal conversion has been observed experimentally by lineage tracing in which progenitor cells are stained and their progeny followed [43-46]. Observations and analysis of intestinal crypt stem cells and their progeny [47,48] are consistent with the conclusion that there is not selection of rapidly dividing progeny clones, but rather that there is turnover of an equipotent stem cell population and that stochastically the progeny of only one stem cell survives [49]. A stochastic model of proliferation of functionally equivalent stem cells, rather than a master stem cell, was shown to result in monoclonality [7,50]. A model of monoclonal conversion that couples cell cycle, cell division, and mechanics of cell movement was used to investigate the consequences of two different scenarios, one in which there is an intrinsically stem-like cell and the other in which there is response of cells to the microenvironment and a population dependent feedback; only the latter produced a stable crypt [51]. Our stochastic model of colon crypt dynamics also reproduces monoclonal conversion by neutral drift.

### Colon cancer

Colon cancer is initiated in adenomatous crypts [52]. These crypts have an altered morphology and an increased number of cells due to mutated cells with an increased probability of proliferating and/or a decreased probability of dying. However, it is not clear where in the crypt the target cells are located [53]. Wright [54] reviewed evidence that is consistent with the initiating mutant cells being located at the bottom of the crypt (“bottom-up” progression), and other evidence that is consistent with the initiating mutant cells being located at the top of the crypt (“top-down” progression). Shih et al. [55] reported that APC mutations, and neoplasia-associated patterns of gene expression, occurred in cells at the top of crypts of small colorectal adenomas, but not in cells at the bottom of the same crypts. Preston et al. [44] reported that monoclonal crypts (of an XO/XY individual) did not show XY or XO adenomatous epithelium growing down into other crypts, suggesting that the target cells for mutations were located in the stem cell region at the base of the crypt. Zhu et al. [56] showed that Wnt activated Prom1 Lgr5 co-expressing cells stem cells developed adenomas, consistent with the idea that neoplastic transformation can be initiated in cells at the bottom of the crypt. Barker et al. [57] detected macroscopic adenomas developing from APC mutant Lgr5 cells located in the stem cell region at the bottom of intestinal crypts. However, they did not detect macroscopic adenomas from APC mutant transient amplifying cells located further up the crypt. Together, these experimental observations suggest that mutant cells further up the crypt may not be capable of yielding adenomas, or may do so with much less efficiency than mutant cells lower in the crypt.

The possibility that mutations in differentiated migrating cells, in addition to proliferating cells, could initiate cancer was supported by the modeling results of Komarova and Wang [58]. Komarova and Cheng [59] provided additional insight using a branching process mathematical model of crypts. They determined that the relative mutation rates of stem cells and non-stem cells, and the relative number of each cell type affect the probability that a crypt containing a mutant cell will progress to cancer. Mirams et al. [60] developed a model to investigate the likelihood that the progeny of a mutated cell will dominate a crypt taking into account cell proliferation and cell adhesion. They concluded that it is unlikely that mutations occurring one or two cells from the base of the crypt will become a dominant clone. Our model has allowed us to investigate the results of locating a mutant cell at the bottom, middle or top of the crypt.

### Chemotherapy

Cancer chemotherapy has the goal of killing abnormal cells while sparing normal cells in order to allow recovery of normal functioning tissue. Mouse and human crypts recover from mild cytotoxic insults [61-63]. Following an exposure to a mild cytotoxic dose of radiation, or a chemical, the number of cells per crypt in mouse tissue has been observed to decrease, then to increase over the initial value, and finally to approach the initial value. The level of decrease and increase, and the time required to recover, are dose dependent [61-63]. The model we describe here displays similar kinetics of recovery. The robust behavior of our model has allowed us to explore the effectiveness of different chemotherapy scheduling protocols.

Exposure to a dose that is effective against the abnormal cells while minimizing life-threatening levels of collateral damage to normal cells might be achieved in two ways, metronomics in which there is a continuous low dose for a prolonged time [64], or fractionated doses in which there are intervals between a maximum tolerated dose to allow repopulation of normal cells between doses [65]. A model in which fractionated doses allow recruitment of quiescent cells into the mitotic cycle after each dose has been described [66].

Improvements in modifying dose-scheduling protocols have been mostly empirical and slow to improve. Human-mouse xenografts and genetically engineered animals [67,68] have been used as surrogates in order to identify differential response to inhibitors of normal and cancer cells [69], and to suggest new schedules of therapeutic doses [70]. Dose fractionation of radiation therapy [71] and dose scheduling of chemotherapy [72] are part of standard clinical protocols. However, deviating from accepted schedules in order to evaluate new protocols is difficult to achieve because of the many different possible combinations of dose intensity and dose scheduling, and the ethical limitation of experimenting on human subjects. Marcu and Harriss-Phillips [73] have reviewed some recent mathematical and computer models of chemotherapy and radiation therapy. Our model of human colon crypts behaves as do normal crypts; it is robust in recovering after exposure to a cytotoxic agent. This has allowed us to investigate the efficiency of a range of scheduling protocols that eliminate abnormal cancer cells and allow recovery of normal crypt function.

### Goals of this study

There were two goals to this study. One was to develop an agent-based model that could simulate the stochastic dynamics of each type of cell in colon crypts. These cell types included stem cells, proliferating cells, and differentiated cells. This model was calibrated by new measurements of cells in biopsies of normal human colon crypts. Simulations indicted that the model had some of the emergent properties of normal crypts, including quasi-stationary stochastic dynamics, monocolonal conversion by neutral drift, formation of adenomas by mutations at the top or the bottom of the crypt, and robust recovery from perturbation. The second goal was to provide the virtual crypt as a tool to conduct simulation experiments *in silico* in order to answer questions and gain insights that could not be addressed by observations of real human crypts in static biopsy specimens. An example of the use of the virtual crypt is presented, *viz.* different chemotherapy protocols were evaluated by simulations with a range of parameter values. The virtual crypt model, developed with an open source application, is being made available to other researchers to carry out further investigations.

## Implementation

### Model description and assumptions

In agent-based models properties are assigned to each agent (biological cell) and then, after computer simulation of repeated cell interaction, a group of cells is produced with emergent properties (crypt) [74]. This agent-based model is in the form of an on-lattice cellular automata model.

In this model, a cell’s properties (probability of cell proliferation, probability of cell death, cell movement, and cell type) are determined by a cell’s position in two gradients along the crypt axis, a divide gradient and a die gradient. The divide gradient determines the probability that a cell will divide; the divide gradient increases from top to bottom. The die gradient indicates the probability that a cell will die; the die gradient decreases from top to bottom.

*Cell proliferation:* when a cell divides, one progeny cell is placed in the same position as the parent cell and the other progeny cell is placed in a position to the right and lower. Placement of one new progeny cell lower than the parent allows the crypt to increase in depth. In order to make space for the this progeny cell, the adjacent cell is pushed to the right and lower. This process is iterated. Lateral placement of one progeny cell to the right or to the left, is necessary, since if successive progeny randomly choose between left and right, then a stable crypt does not result, as can be seen in simulations by toggling off “Polar Division”. Right, rather than left, has been chosen arbitrarily. Such placement of one progeny cell laterally has been a feature of other models [33,75]. If a new cell reaches the right edge of the roll-out image of the crypt it will reappear on the left.

*Cell movement:* when a cell dies, cells below are pulled up into the empty space. This results in cells moving up in a vertical column. Cell movement is not the result of a cell being pushed up after cell division. This is consistent with the observations that cell movement continues in the absence of cell division [33,76].

**Figure 1:**
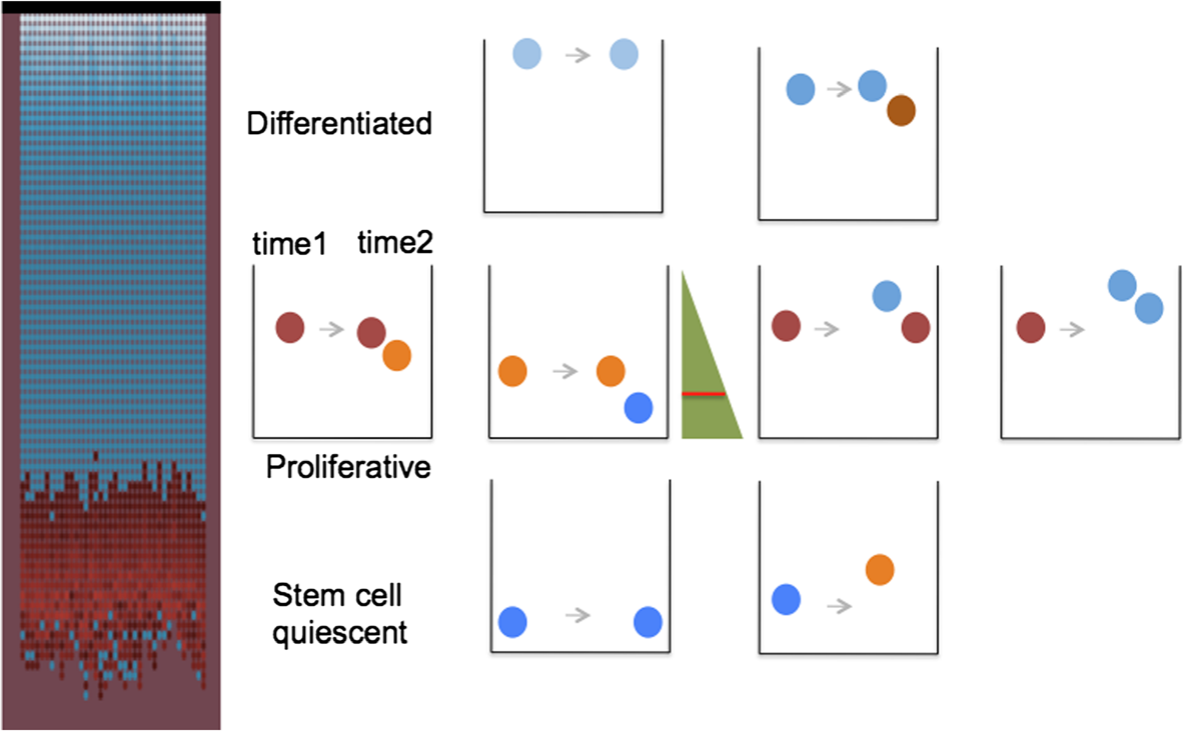
**Cell type is determined by a cell’s position in the divide gradient.** The virtual crypt is shown on the left. Non-proliferating cells are blue, proliferating cells are red. The various cartoons of crypts on the right illustrate the various possible locations of progeny cells after cell division at time 2, and the cell type of the progeny. The cell type of the progeny cells is determined by their position in the divide gradient (green triangle). Below the red line in the green triangle is the region of quiescent cells. In the top row of cartoons, a differentiated cell (blue) at the very top of the crypt may remain undivided at time 2; or a differentiated cell lower in the crypt may divide and one progeny may become a proliferative cell (red). In the center row of cartoons, the progeny of a proliferative cell (red) that arrives low in the gradient may become a quiescent stem cell (blue); or if it arrives high in the gradient may become a differentiated cell (red). In the bottom row of cartoons, a quiescent stem cell (blue) may arrive in the region of proliferating cells and become an active stem cell (red). Note the possibility of bidirectional conversion of cell types.

*Cell type:* a cell’s type is determined by its position in the divide gradient, Figure 1. A progeny cell that has moved away from the position of its parent will experience a different gradient value than its parent, and as a consequence may have a different phenotype than its parent. For instance, the progeny of a differentiating cell may move down into the region of a proliferating cell and become a proliferating cell. The progeny of a proliferating cell may move down into a niche region of quiescent stem cells. In the niche it responds to a quiescent gradient, not the divide gradient. A quiescent stem cell may, if cells above it are removed, move into the region of proliferating cells and become an active stem cell.

Cell division and cell death are discrete events and result in a stochastic change in the number of cells per crypt as a function of time. Such a stochastic system may be subcritical and go extinct, or be supercritical and increase without bounds, or may be critical and display quasi-stationary dynamics with the number of cells increasing and decreasing about a mean value, over time. The number and variation of cells per crypt, and the number and variation of each type of cell, measured in a series of human crypts, suggests that biological crypts are in a critical state. One challenge of modeling is to determine critical parameter values for which the model behaves with realistic quasi-stationary dynamics. That is, with an average number of each cell type, and the variation of cell types measured in real human colon crypts.

### Model instantiation

The crypt model was produced with the NetLogo v.4.3.3 application. NetLogo is a multi-agent programmable modeling environment. It is authored by Uri Wilensky and developed at The Center for Connected Learning (CCL) and Computer-Based Modeling. It is a multi-platform (Mac or Windows) open-source application available at http://ccl.northwestern.edu/netlogo/.

**Figure 2:**
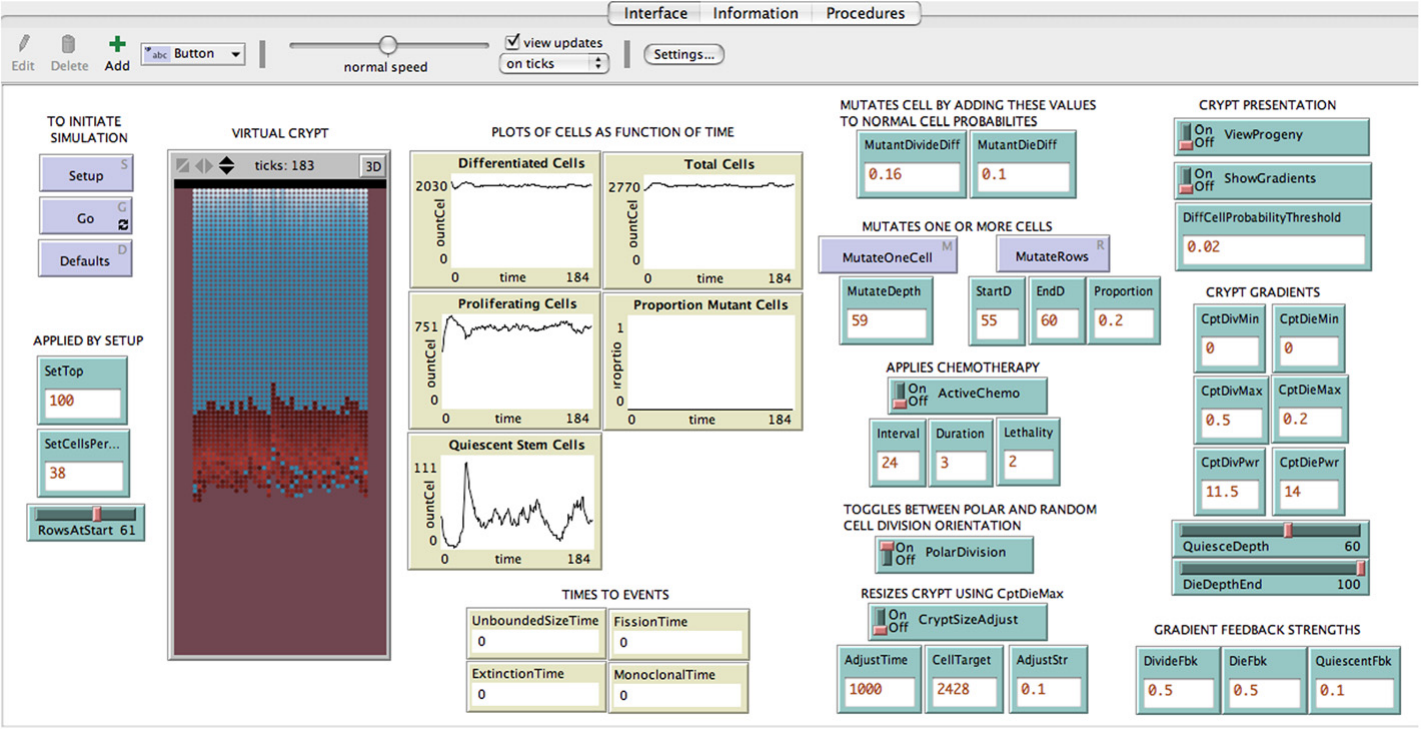
**Interface of the virtual crypt model implemented in the application NetLogo.** The crypt cells appear in the vertical rectangle at the left, proliferating cells in red, and non-proliferating cells in blue. Blue boxes initiate events. Green boxes and sliders allow user to input parameter values at the interface. Beige boxes display simulation outputs of cell numbers as dynamic plots, or single digital values of the time when an event has occurred.

The virtual crypt model is included as Additional file 1. It can be run from the graphical user interface after downloading the NetLogo application, as seen in the Additional file 2. The model includes three parts, (1) Interface, Figure 2, (2) Information (text with model details including inputs, gradients, outputs, and parameter sweeping), Additional file 3 and (3) Procedures (computer code and comments), Additional file 4.

The Interface screen, Figure 2, displays the movement of cells in a virtual crypt, and numerical outputs. The outputs include dynamic plots of cell numbers as a function of time and single values for the time to complete specific events. Output can also be exported as numerical values in a spread sheet format, useful for parameter sweeps and statistical analysis. The Interface allows a user to input different numerical parameter values without changing the computer code.

The virtual crypt is a two dimensional “roll-out” representation of the cup-shaped three dimensional biological crypt. The cells exist on a two-dimensional grid. The width and height of the crypt are input using the input boxes “SetCellsPerRow” and “RowsAtStart”. Upon activating the “Setup” and “Go” buttons, the cells appear in the virtual crypt and respond to the divide gradient and die gradient.

Non-dividing cells appear in blue and proliferating cells appear in red. The quiescent stem cells appear in blue at the bottom of the crypt. The proliferating cells appear in red above the quiescent stem cells. The differentiated cells appear in blue above the proliferating cells. The regions occupied by the quiescent stem cells, proliferating cells, and differentiated cells are determined by values input with the “QuiesceDepth” slider, and the “DiffCellProbabilityThreshold” input box.

After the “Go” button is pressed, the cells divide, move, and die. The number of each cell type changes with time as each cell experiences different regions of the divide gradient, and the crypt evolves. The number, as a function of time, of each cell type appears in dynamic plots.

Divide and die gradients can be viewed by turning on the toggle “ShowGradients”. The shape of the gradients can be altered by changing the input values of the crypt maximum, crypt minimum, and the crypt power. Other inputs allow modifying parameter values that affect mutations, chemotherapy, cell division orientation, crypt size, and gradient feedback delays.

### Calibration of model with measurements of biopsy

Calibration of model parameters involved three steps: (1) measurement of the number of each of the three cell types in a series of crypts in human biopsy specimens, in order to determine the average number of each cell type and the variation of each cell type between different crypts; (2) parameter sweeping to determine the value for the shape of the divide gradient and the shape of the die gradient that reproduces the measured average number of each cell type; and (3) parameter sweeping to determine the value for the strength of the cells’ response to the gradients that would reproduce the measured variation of each cell type.

(1) Measurement of the number of each cell type in human crypts

**Figure 3:**
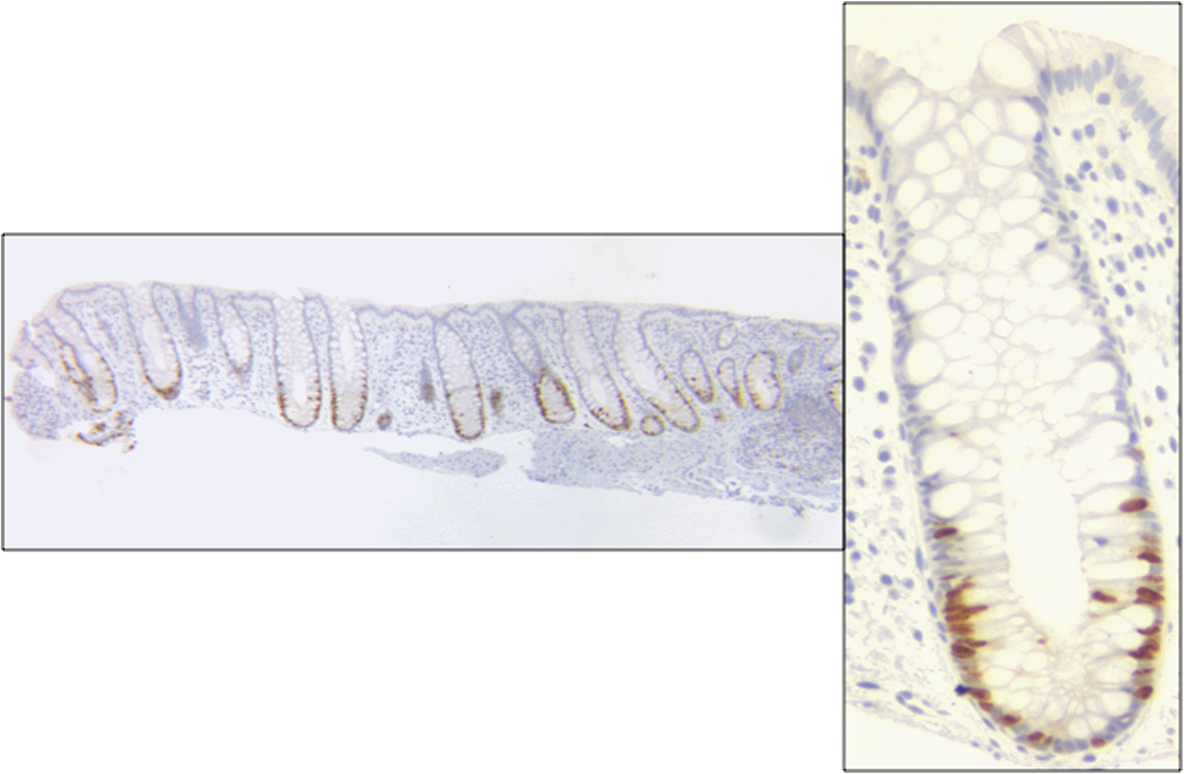
**Section of biopsy of normal human colon stained brown with antibody to proliferating antigen Ki-67, and counterstained blue with hematoxylin.** Left, example of several adjacent crypts in the tissue. Right, enlargement of a single crypt showing brown stained proliferating cells, blue stained quiescent stem cells at the bottom of the crypt, and blue stained differentiated cells in the top two-thirds of the crypt.

Three different cell types can be recognized when crypts of human biopsy specimens are stained with an antibody to the proliferating cell antigen Ki-67, Figure 3. Proliferating cells near the bottom one third of the crypt are stained brown. Proliferating cells include cells that have been called active stem cells and transient amplifying cells. Differentiated cells, which are not proliferating, located in the top two-thirds of the crypt, are counterstained blue. Quiescent stem cells, which are not dividing, located at the bottom of the crypt, are counterstained blue. Each of the three different cell types were measured in 49 crypts. This was the total number of crypts which were available. Details of the experimental methods are given in Additional file 5. The mean, standard deviation, and coefficient of variation (C.V. = standard deviation/mean) are given in Table 1. The C.V. allows comparison of the variation between different cell types with different mean values. It is noteworthy that the C.V. of the quiescent stem cells is large, 102%, as might be expected of a stochastic variation about a small mean.

(2) Determining the parameter values that control the shape of the divide and die gradients in order to reproduce the average of each cell type in human crypts

The crypt gradients that affect a cell’s probability to divide or die would be expected to influence the number of each cell type and the total number of cells in a crypt. The shape of the divide gradient is determined by the value of the input parameter “Crypt Divide Power”. This value can be changed by using the input box labeled “CptDivPwr”, as shown in Figure 2. The gradient is *p* = *y*^*n*, where *p* is the probability that a cell will divide, *y* equals 1 at the bottom of the bottom of the gradient and 0 at the top, and *n* is the power to which y is raised. This formula allows different shape gradients to be generated, *n* = 0 is no gradient, *n* = 1 is linear, *n* > 1 is concave. The greater the value of *n*, the more extreme is the concavity. This is demonstrated in the video in Additional file 6.

The ranges of parameter values were investigated (parameter sweeping) using the Behavior Space function in NetLogo. A value of “Crypt Divide Power”, *n* = 11.5 was chosen to simulate the observed mean number of each cell type, and mean total number of cells. Similarly, the value of “Crypt Die Power”, *n* = 14 was chosen. The probability that a cell will die is 1 at the top of the gradient, and 0 at the bottom of the gradient. These values were used to simulate 49 different crypts, each initiated with a different size, and allowed to run for 1000 time units.

**Figure 4:**
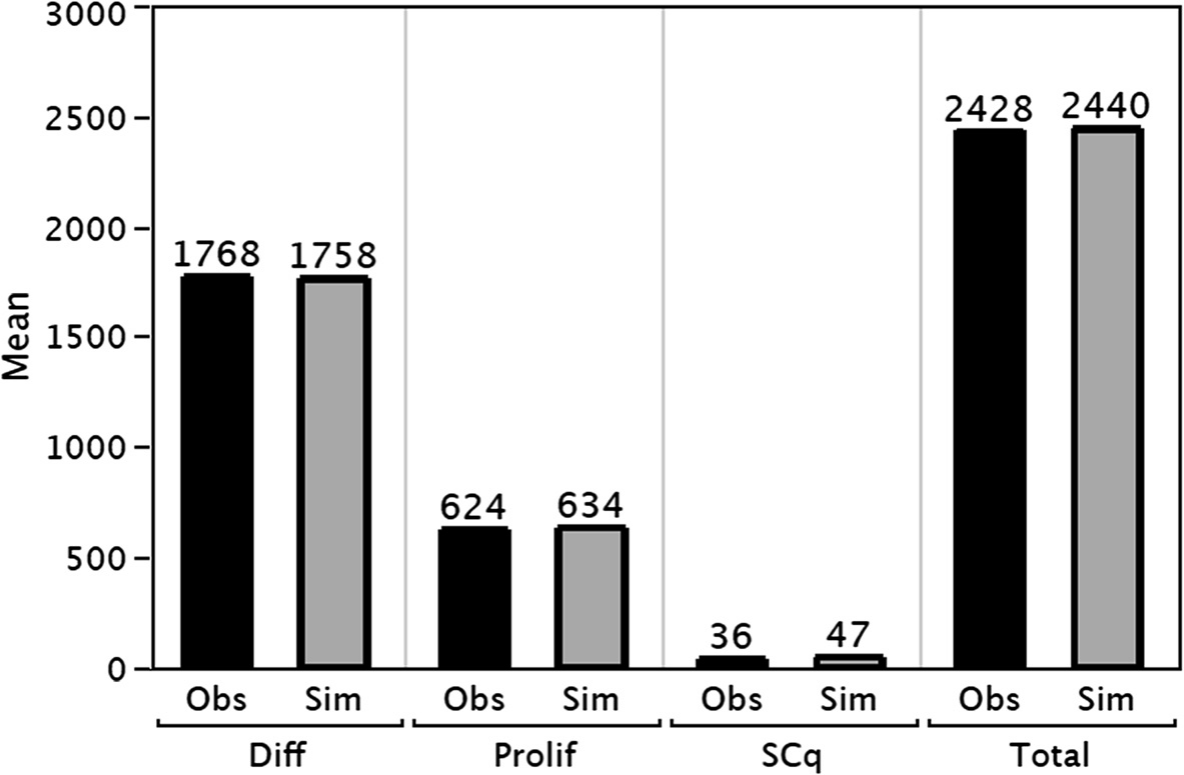
**Comparison of the observed (Obs) mean number of each cell type and mean number of total cells per crypt measured in 49 crypts, with the simulated (Sim) numbers.** Diff = differentiated cells, Prolif = proliferating cells, SCq = quiescent stem cells.

For each cell type the observed mean values for 49 human crypts is shown in Figure 4 together with the mean values for 49 simulated crypts. The simulated number of each cell type is not significantly different than the observed number of each cell type (Chi-squared test, p >0.05). This suggests that the model, with appropriate parameter values for the gradients, produces “virtual crypts” that resemble observed human crypts, for the average number of each cell type, and for the average total number of cells.

**Table 1:** Measured number of each cell type per crypt

**Cell type**	**Mean**	**S.D.**	**C.V. %**
Quiescent stem cells	35.7	36.3	102
Proliferating cells	623.9	234.1	37.5
Differentiated cells	1768.2	434.5	24.6
Total	2427.8	504.4	20.8

Note: Values are for counts of 49 different crypts. Proliferating cells include transient amplifying cells and active stem cells. S.D standard deviation, C.V coefficient of variation.

(3) Determining the parameter values of each cell’s response the gradients in order to reproduce the variation in numbers of each cell type in human crypts

The number of each kind of cell in a biological crypt would be expected to vary with time with quasi-stationary dynamics, *i.e.* varying in time stochastically about an average value. The variation of a single crypt over time can be inferred from the measurements of the variation of multiple human crypts at a single time.

The values chosen for the divide gradient and die gradient were adequate to simulate the measured average number of each cell type and the measured average total number of cells per crypt. However, these values were not adequate to simulate the measured variation in the number of each cell type and the measured variation in the total number of cells per crypt. For instance, whereas the measured C.V. of the total number of cells was 20.8%, the simulated C.V. was only 3.3%. There was also a difference between the measured and simulated C.V. for each cell type. Most notably, for the quiescent stem cells the measured C.V. was 102%, and the stimulated C.V. was only 44.3%.

Quiescent stem cells that move out of the their niche can become active stem cells. Active stem cells above the niche are in gradient values that determine a high probability of dividing. Some of the progeny of active stem cells may move back down into the niche and become quiescent stem cells. Therefore, the number of quiescent stem cells could vary stochastically over time. The quiescent stem cell population with the large variation of 102% and small average number of 35.7 has a danger of going extinct. However, extinction might be avoided if the rate of change in cell type is influenced by feedback, a delay in a cell’s response to the value of the gradient in which it is relocated.

In this model a cell that moves to a new position in the divide gradient will acquire a new probability of dividing immediately if the gradient feedback strength = 1. If the gradient feedback strength <1, the cell will acquire a new probability of dividing, at each time unit, that is intermediate between its probability of dividing at its old position and its new position. This will result in a delay in responding the gradient. If the gradient feedback strength = 0, the cell will not respond to the gradient in its new position and retain the probability of dividing that it had before it moved.

**Figure 5:**
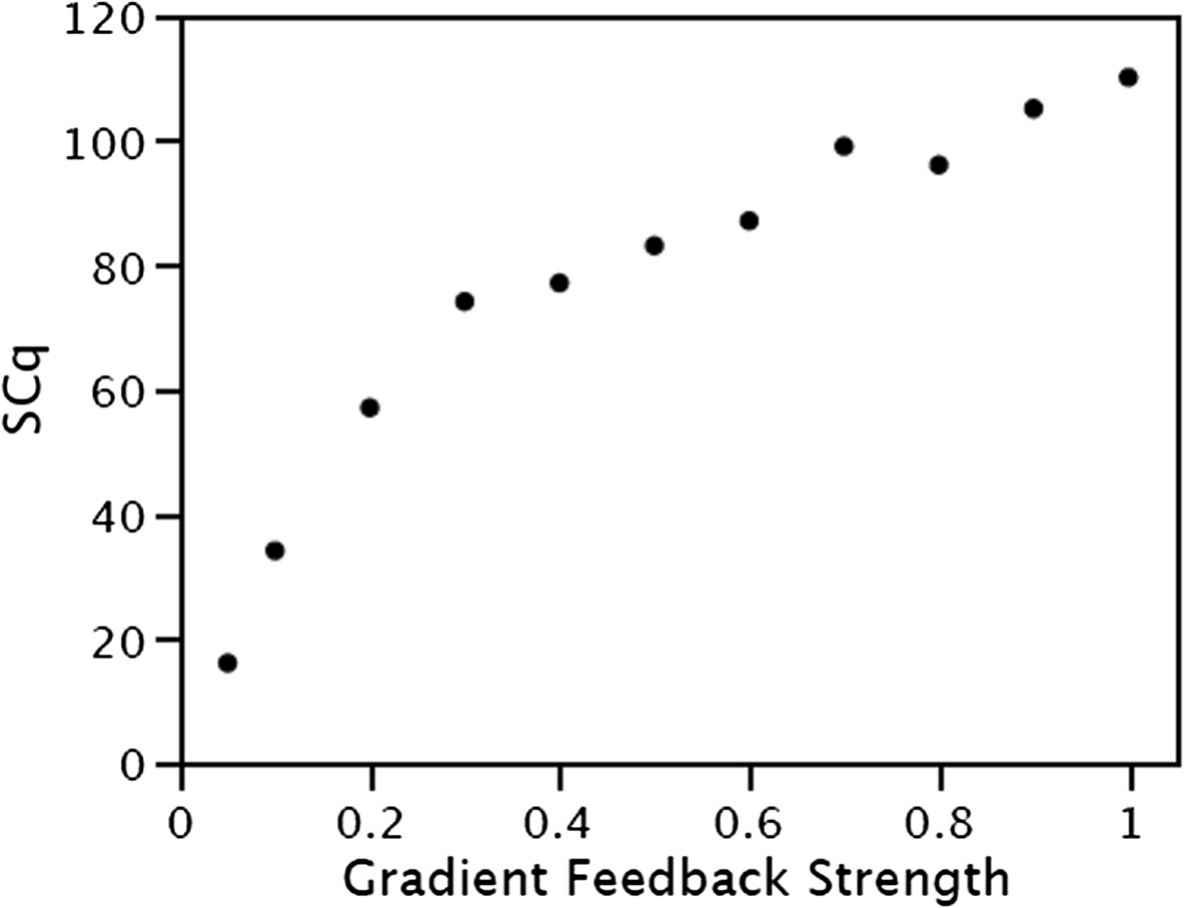
**Effect of the quiescent gradient feedback strength on the number of simulated quiescent stem cells (SCq).** Gradient feedback strength = 1 produces an immediate response, gradient feedback strength = 0 produces no response, and gradient feedback strength < 1 produces an intermediate response.

**Figure 6:**
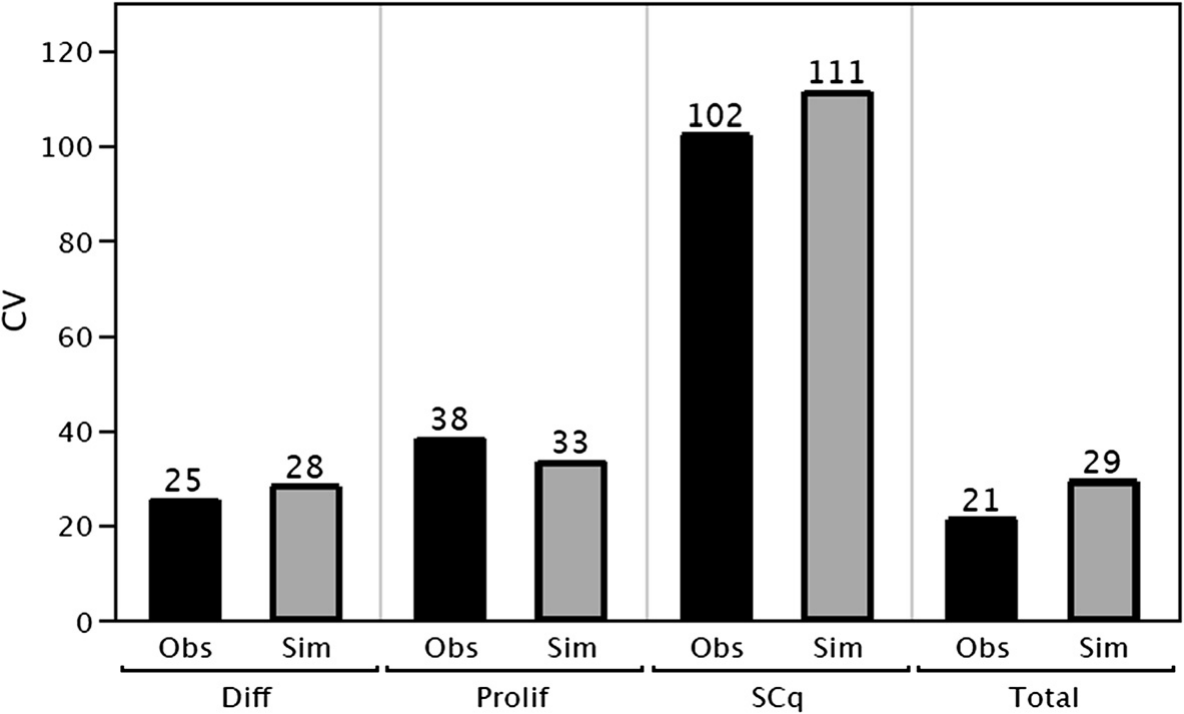
**Comparison of the observed (Obs) and simulated (Sim) coefficient of variation of number of each cell type, and coefficient of variation of number of total cells per crypt.** Diff = differentiated cells, Prolif = proliferating cells, SCq = quiescent stem cells.

The effect of gradient feedback strength on the average number of quiescent stem cells was determined for a range of gradient feedback strengths, Figure 5. With a gradient feedback strength = 1 (no delay in responding the gradient) the simulated average number of quiescent stem cells is greater than the measured value. With a gradient feedback strength = 0.1 (a modest delay in responding to gradient) the simulated average number of quiescent stem cells is similar to the measured average number of quiescent stem cells. And notably, the simulated coefficient of variation of the number of quiescent stem cells is similar to the observed value, Figure 6. These results suggest that quiescent stem cells that move outside of their niche, into the region of the proliferating cells, will respond to the gradients with some delay before become active stem cells.

Proliferating cells and differentiated cells also respond to their relocated position in the divide gradient with a feedback delay. The observed coefficient of variation of each cell type, can be simulated with delayed response to the gradient, Figure 6. This is accomplished by setting their gradient feedback strengths < 1. Since a delay is required to simulate the measured variation in the total number of cells per crypt and the measured variation in the number of each cell type, these results suggest that proliferating and differentiated biological cells may also respond to the gradients with a delay.

### Verification of model behavior

The behavior of the calibrated crypt model was verified by its ability to simulate three emergent properties observed in biological crypts, (1) monoclonal conversion by neutral drift, (2) adenoma formation by mutants at different locations, and (3) robust recovery from exposure to a cytotoxic agent.

(1) Virtual crypts undergo monoclonal conversion by neutral drift

Crypts can undergo monoclonal conversion, in which all cells in the crypt are the product of a single cell. Monoclonal conversion may be the result of one stem cell producing progeny that outcompete the progeny of other stem cells; or may be the result of only one stem cell at a time producing progeny [77]. Experimental observations are consistent with neutral drift in which the progeny of only one stem cell survive, without the need for a proliferative advantage of the progeny of that stem cell [47,48].

Monoclonal conversion was visualized in the virtual crypt by switching on the toggle “ViewProgeny”. No cells were given an proliferative advantage. Initially, different cells appear with different colors, but over time all the cells in a crypt have a single color, the progeny of one cell. This can be seen in Additional file 7. Each repeat of the experiment generates a crypt of a different color and a different time to achieve monoclonality. The distribution of times are approximately log normal with a mean of 2633 time units and standard deviation of 1610. These simulation results indicate that the virtual crypt behaves similarly to biological crypts, *i.e.* they undergo monoclonal conversion by neutral drift, and that selection is not necessary.

(2) Virtual crypts initiate adenomas by both “top-down” and “bottom-up” mutations

Abnormal proliferation of cells in crypts can lead to colorectal adenomas, crypts with more cells than normal. Some observations have been interpreted as suggesting that the mutant progenitor cells are located at the top of the crypt resulting in “top-down” morphogenesis [55]. Other observations have been interpreted as suggesting that the progenitor cells are located at the bottom of the crypt resulting in “bottom-up” morphogenesis [44]. The virtual crypt was used to investigate the effects of placing mutant progenitor cells at different positions in the crypt. Mutant cells were generated with a 1.3× greater probability of dividing than normal cells, and with a 1.1× greater probability of dying. The efficiency of forming adenomas, considered as crypts that have 40% more cells than normal, was determined.

The results in Table 2 indicate that adenomas can be formed by mutant cells initially located in each of several different regions of the crypt. Mutant cells placed at the bottom of the crypt in the region of quiescent stem cells, or placed about 1/3 up from the bottom of the crypt in the region of proliferating cells, or placed about 1/3 from the top of the crypt in the region of the differentiated cells, each formed adenomas, but with different efficiencies. These results suggest that both “top-down” and “bottom-up” morphogenesis may occur, but that “bottom-up” is more efficient. The most efficient location is within the lower region of proliferating cells. This would be expected to include active stem cells. These simulation results indicate that virtual crypts behave similarly to biological crypts, *i.e.* they can form adenomas from mutant cells located at the top or the bottom of the crypt.

(3) Virtual crypts are robust and recover after a cytotoxic dose of chemotherapy

Crypts have been reported to recover from cytotoxic chemotherapeutic drugs and ionizing radiation in a dose dependent manner. After a single dose to mice the total number of cells per crypt decreases, overshoots the initial value, and then returns to the initial value, with the rate of recovery dependent on dose [61]. In order to determine if the virtual crypt was similarly robust in response to cytotoxic perturbation, a virtual crypt was exposed to a single dose and the total number of cells per crypt was followed as a function of time.

**Figure 7:**
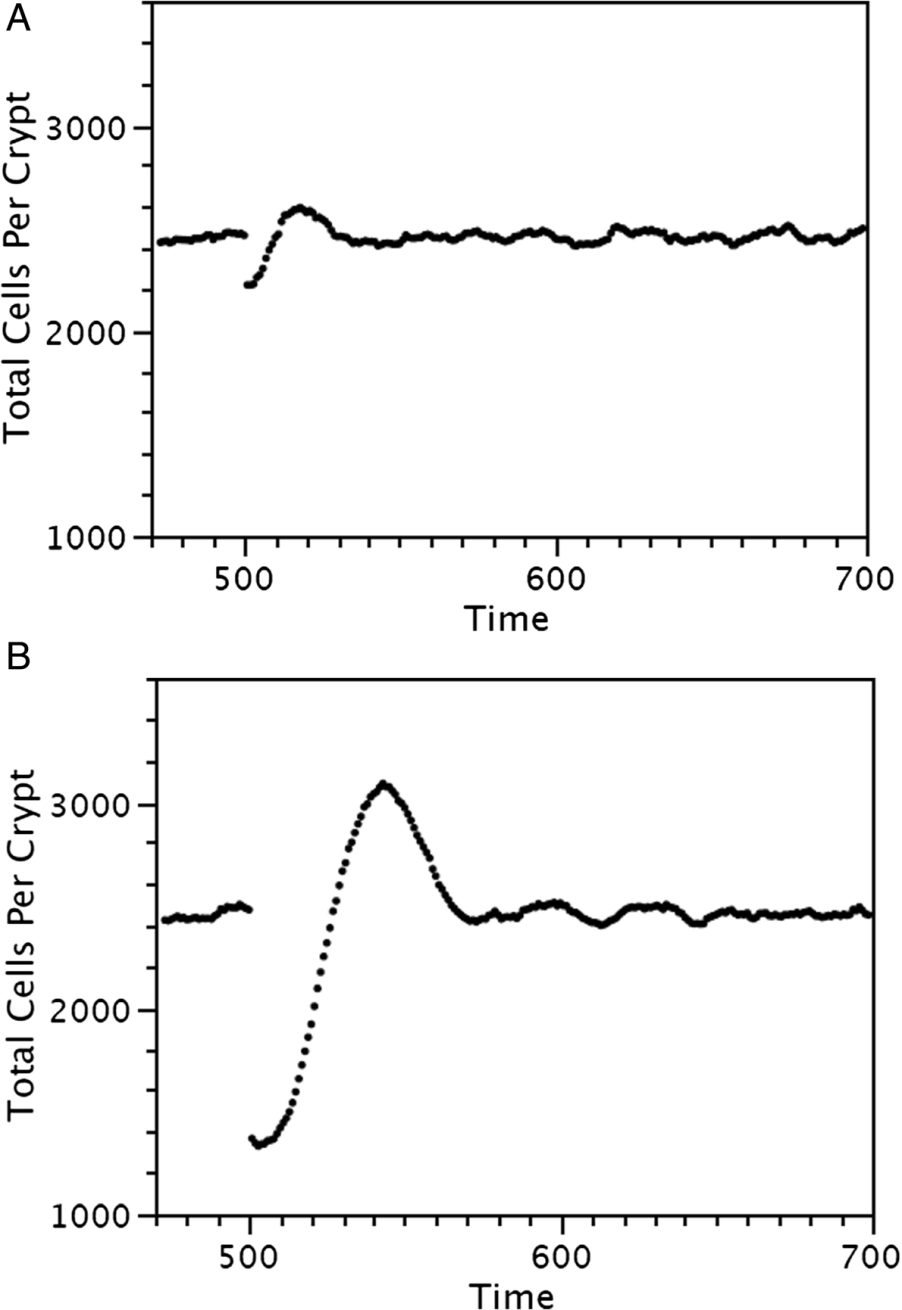
**Recovery of virtual crypts after cytotoxic therapy.** Crypts with about 2400 cells were exposed at time 500 to a low dose of cytotoxic therapy **(A)**, or high dose of cytotoxic therapy **(B)**.

The probability that a cell in a virtual crypt will die after cytotoxic chemotherapy is its probability of dividing before chemotherapy times the “Lethality” of the dose, *i.e.* more rapidly dividing cells are more sensitive to a specific cytotoxic dose. Virtual crypts were exposed to a “Lethality” of 5 or 320, and the total number of cells per crypt was determined as a function of time, Figure 7. The virtual crypts recover with kinetics similar to those that have been reported for biological crypts, the total number of cells decreases, overshoots the initial value, and then returns to the initial value. In addition, there is a dose dependent minimum number of cells, maximum number of cells, and time to recovery. These simulation results indicate that the virtual crypts are robust, and that they behave similar to biological crypts in their response to a cytotoxic agent, both in kinetics and in a dose dependent manner.

**Table 2:** Top-down and bottom-up mutations each produce adenomas

**Position of mutation (cell rows from top)**	**Mutant cell type**	**Efficiency %**
Top third (20)	Differentiated cell	8.5
Lower third (59)	Proliferating cell	85
Bottom (65)	Quiescent stem cell	57

Note: Efficiency is the percent of 200 crypts that achieved a size 40% larger than normal crypts in 500 time units.

Because the calibrated virtual crypts recovered from cytotoxic doses they could be to be used to simulated cancer chemotherapy and to explore the effectiveness of different dose schedules.

## Results

In order for a computer model to be useful as an informative simulation tool it needs to be calibrated to the real system that it intends to simulate, shown to reproduce the behavior of the real system, and accessible as a tool to simulate new scenarios and produce interpretable quantitative results. This section provides an example that shows that the virtual crypt model satisfies the third criterion, *i.e.* that it can be used to simulate a range of chemotherapy scenarios and can produce informative results.

A desired chemotherapy protocol might be one that (1) removes all tumor cells, (2) uses a minimum duration of exposure to chemotherapy, and (3) simultaneously minimizes collateral damage to healthy non-tumor cells. The model was used to determine the effect of a range of parameter values. Several parameters of chemotherapy can be modified from the graphical user interface. These include, the Lethality (increased probability of dying), Duration (time period of exposure), and Interval (period between the beginning of one period of exposure and the beginning of the next). Fractionated chemotherapy, that allowed recovery between doses, was simulated in order to answer the question “What duration of chemotherapy would be optimal?”

**Figure 8:**
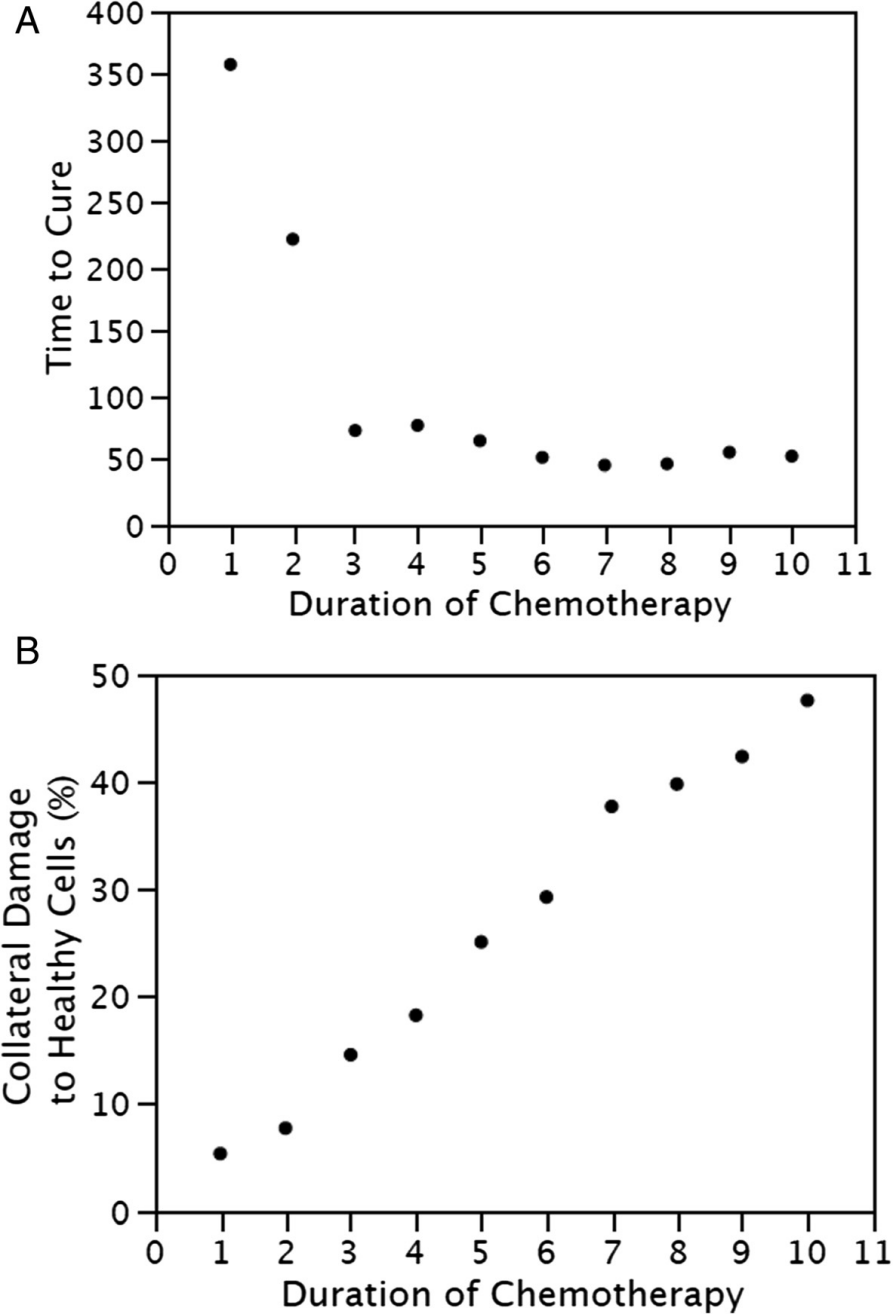
**Simulation of chemotherapy protocols.** The effects of different durations of chemotherapy on time to eliminate all tumor cells **(A)**, and the collateral damage to healthy cells **(B)**. A duration of chemotherapy of 3 time units eliminates all tumor cells while minimizing collateral damage to healthy cells. Each Duration time unit is 4.5 hr.

Mutant tumor cells were simulated by adding 0.16 to their probability of proliferating, and adding 0.1 to their probability of dying. They were placed in the lower third of the crypt and allowed to proliferate to form an adenoma, Additional file 8. When the number of mutant tumor cells reached 50% of the total cells in the crypt, a low dose of chemotherapy was applied, Lethality = 2. The Intervals between doses were fixed at 8× the Duration of doses. For a range of chemotherapy Durations, the time to eliminate all mutant tumor cells (Time to Cure) is shown in Figure 8, **A**; and the collateral damage to healthy cells is shown in Figure 8, **B**. The longer the Duration of chemotherapy the shorter the time to eliminate all mutant tumor cells (Time to Cure). However, a longer Duration of chemotherapy also increases the collateral damage to healthy cells. Comparison of the two graphs suggests that a Duration of chemotherapy of 3 time units would be an optimum trade off. This Duration of exposure to chemotherapy would be sufficient to eliminate all tumor cells while minimizing collateral damage to healthy cells. A longer Duration of exposure to chemotherapy would have about the same time to cure, but would increase the collateral damage to healthy cells. The simulated kinetics of mutant cell death and normal crypt recovery during chemotherapy are shown in the video in Additional file 9.

These simulation results indicate that the robust virtual crypt model can be used for *in silico* experiments to investigate different chemotherapy scheduling protocols and produce quantitative results. The relationship between the range of Duration times and the balance between time to cure and collateral damage to healthy cells would not be easily obtained by other means.

## Discussion

The model presented here was developed to simulate the stochastic cell dynamics in crypts of the human colon. It took into account recent evidence that cell types are plastic, *i.e.* that there can be inter-conversion between cell types. The model is the first to reproduce the average number of each cell type (stem cells, proliferating cells, and differentiated cells) and the variation of each cell type, measured in human colon crypts. It also reproduced the behavior of biological crypts, including monoclonal conversion by neutral drift, formation of adenomas by initiating mutations at various locations along the crypt axis, and robust recovery after exposure to a cytotoxic agent. The utility of the model was demonstrated by experiments conducted at the graphical-user interface that explored the effect of a range of parameter values for chemotherapy.

### Inter-conversion of cell types

In this agent-base model, progeny cell types are determined not by the type of their parent, but rather by their position in a divide gradient along the crypt axis. The divide gradient has a low value at the top of the crypt and a higher value toward the bottom of the crypt. A progeny cell that moves to a new position away from a parent has a type that is determined by its new position in this gradient, rather than by the type of its parent, Figure 1. The value of a gradient at a cell’s position along the axis of the crypt depends upon the length of the gradient, that is, the total number of cells in the crypt. As the total number of cells stochastically increases by division or decreases by cell death, the value of the gradient at a position may change. A progeny cell may arrive in a higher or lower gradient value than its parent. As a consequence, the change in cell type from parent to progeny is not limited to a change in one direction, but may be bidirectional. For instance, a proliferating cell may produce one or more progeny cells that are also proliferating cells, or one or more progeny cells that are differentiated cells, or one or more cells that are quiescent stem cells. Similarly quiescent stem cells may produce proliferating cells; and the reverse, proliferating cells may produce quiescent stem cells.

### The divide gradient

After cell division, a progeny cell’s type will depend on its response to the value of the divide gradient at its new position. In this model the response is determined by the “Gradient feedback strength”. It is the response of a progeny cell to the difference in the gradient value at its new position compared to the old position of its parent. The strength of the response affects the variation of cell numbers over time. Parameter sweeping was used to determine value of the “Gradient feedback strength” that reproduced the observed coefficient of variation between crypts, Figure 6.

The divide gradient may be determined by the concentration of a ligand along the crypt axis. The response to the divide gradient may be influenced by the number of receptors on the cell, as well as the time period required for signal transduction, gene expression, protein synthesis, protein processing, and protein translocation to the cell surface. These, in turn, may depend on the rate of progression through the cell cycle. Molecules with gradients along the crypt axis have been described [18,37-39]. Some of these molecules have been the basis of other crypt models. These molecules include Wnt [78], Wnt and Notch [8,32], and EphB receptors and ephrinB ligands [79]. Our model is agnostic as to the identification of the molecule, or combination of molecules, that might be responsible for the divide gradient and the cell’s response to the gradient.

### The die gradient

Homeostasis requires that cells be removed as new cells are produced. The die gradient along the crypt axis indicates the probability that a cell will die, and be removed. There is a greater probability that a cell will be removed at the top than at the bottom. The consequence of a cell near the top being removed is that a cell below it can move up into its former position. This process can be iterated, resulting in cells moving up the crypt in a column. Kaur and Potten [76] observed that cell migration occurred after recovery from radiation, hydroxyurea, or other cytotoxic agents, even in the absence of mitotic activity. This is consistent with cell movement resulting from cells being pulled up from the top, rather then being pushed up from the bottom. It is not clear what is the mechanism of cell removal. Apoptosis, and removal of dead cells by macrophages, is most frequent at the top [80]. Cell removal may be a response to the curvature of the cell layer at the lumen, as seen in Figure 2. This could result in removal of densely packed cells and death by anoikis [81]. However, exfoliation, common in neoplasia, has been reported to be rare in normal human colon crypts [82]. Alternatively, the die gradient may be the result of a cell division counting mechanism, such as loss of telomeres [22]. This model does not invoke a specific mechanism for cell death and cell loss.

### Stem cells

We measured an average of 35.7 quiescent stem cells per human colon crypt. This was determined as the number of cells in the bottom of the crypt that did not stain with the antibody to Ki-67, a protein that is expressed in proliferating cells [83]. Other investigators have identified quiescent stem cells, active stem cells, or stem cell precursors, as cells at or near the bottom of the crypt that uniquely express specific proteins. These proteins include *Lgr*5 [84], *Bmi1*[85], musashi-1 [86,87], aldehyde dehydrogenase 1 [88], and *Lrg1*[89]. Stem cells have also been recognized by utilizing functional assays [29,90] or by linear tracing [45]. Potten et al. [91] measured the number of clonogenic cells after radiation in the small intestine of mice and concluded that there were about 32 “functional” stem cells. Loeffer et al. [33] developed a model of steady-state proliferation in mouse intestine crypts and by fitting successive labeling index data concluded that there are more than 4 and more likely closer to 16 stem cells per crypt. Kozar et al. [92] calculated the number of stem cells per crypt in the mouse small intestine as 5–6, and in the colon as 7, by lineage tracing of crypts with spontaneous mutations in [CA] 30 repeats. Nicolas et al. [93] analyzed methylation patterns of human colon crypts and concluded that there was a modal value of between 15 and 20 stem cells per human colon crypt. 

The differences in the estimates of the number of stem cells per crypt may be due to several factors, including differences in the types of cells that are detected by different assays, *i.e.* quiescent stem cell, active stem cells, or stem cell precursors. Other contributing factors include differences in the size of mouse and human crypts, and differences in size of crypts in the intestine and in the colon.

We measured the variation of the number of quiescent stem cells in adjacent crypts in a fixed biopsy specimen and found a large coefficient of variation, 102%. This variation in different crypts at one time suggests that the number of quiescent stem cells in any one crypt is dynamic and varies over time [94]. Our model reproduces this variation of the number of stem cells. It also reproduces the variation of each of the other cell types measured in human crypts. This was achieved by determining appropriate values for the strength of feedback response of cells to their gradients. Feedbacks have been included in other models of the dynamics of crypts [5,32,95,96] and other similar structures [31,97]. These models have included feedbacks in order to simulate an average number of cells over time, whereas in our model feedbacks were included in order to simulate the variation in number of cells over time.

### The virtual crypt is robust to perturbation

The virtual crypt is robust, as are biological crypts, in recovering after exposure to a cytotoxic agent, Figure 7. When unperturbed, the number of cells per crypt is in a quasi-stationary state. After a crypt is exposed to a cytotoxic dose there is a reduction in the total number of cells, mostly by reduction in the proliferating cells which are more sensitive than the quiescent cells which are non-dividing and the differentiated cells which have a low probability of dividing. The reduction in the total number of cells has the effect of quiescent stem cells arriving within the region of the divide gradient. This results in them having a greater probability of dividing. The former quiescent stem cells convert to become active stem cells and their progeny become proliferating cells. The result is that the total number of cells increases briefly until the quasi-stationary state is restored. Similar recovery behavior has been achieved in other crypt models [8,98].

### The virtual crypt model was used to investigate chemotherapy protocols

Since the virtual crypt recovers after exposure to cytotoxic agents, it can be used to simulate chemotherapy, or radiation therapy, with different scheduling protocols. Differences in the interval between cytotoxic doses, the duration of the doses, and the lethality of each dose can be varied at the graphical user interface. In one example, parameter sweeping was used to determine the optimum duration of chemotherapy that would eliminate all mutant tumor cells with the minimum collateral damage to healthy cells, Figure 8. The response of the virtual crypt to chemotherapy as a function of time can be seen in the video demonstration provided in Additional file 9.

There are limitations in translating the results of simulating cancer chemotherapy protocols. It would be necessary to determine the difference in the rates of cell proliferation, cell death, and cell differentiation of the mutant cells from those of normal cells. Proliferation and death rates are routinely measured in cell culture, albeit not in tissue context. Cell differentiation rates can be measured by utilizing the proportion of clonal populations that stain positively for expression for protein markers of differentiation [99]. More stringent limitations are that the model does not yet take into account angiogenesis, invasion, and the effect of cytotoxicity on other tissues such as bone marrow or heart. Although no model is complete, this model and other *in silico* models, have provided insights that are not readily available by other means [73].

### Additional applications of the virtual crypt model

One of the goals of this project was to provide a tool that would facilitate *in silico* experiments. Several examples of experiments using the virtual crypt model have been described in the sections “Verification of model behavior”, and “Results”. The model of the virtual crypt is being made available for other users to conduct additional *in silico* experiments, Additional file 1. The experiments can be conducted by inputting single parameter values at the graphical user interface and seeing the output plots in real time, or by conducting parameter sweeps using the Behavior Space tool and collecting numerical data in table or spreadsheet format for statistical analysis. Some example additional experiments include the following:

#### Modeling crypts of other organisms

The model described here was calibrated with measurements of the number of cells in a human biopsy. Crypts of other organisms, such as the mouse for which there is a large amount of experimental evidence, could be modeled by turning on the “ResizeCryptAdjust” toggle and entering the appropriate number of cells per crypt in the “CellTarget” box.

#### Investigating non-homeostatic conditions

Parameter values were determined that allowed the virtual crypt to be in a critical state, *i.e.* to maintain quasi-stationary numbers of each cell type over time. However, a stochastic process may be critical, subcritical, or supercritical depending on the ratio of cell birth and cell death. This ratio may be altered by mutation, cytotoxic therapy, or redistribution of growth factors. The result could be crypts becoming either extinct or unbounded in cell number. The reporter box “ExtinctionTime” will display the time when a crypt becomes extinct, and the reporter box “UnboundedSizeTime” will display the time when a crypt becomes unbounded in cell number. Multiple runs using the Behavior Space tool can provide information on the distribution of times for such events, and the range of parameters for which the crypt is critical, subcritical, or supercritical.

#### Exploring modes of therapy

Fractional chemotherapy was described as an application of the crypt model. In fractional chemotherapy there is a repeated application of a maximum tolerated dose of a cytotoxic agent for fixed duration, interspersed with a period of no exposure to the agent to allow recovery. Other kinds of therapy can be explored with the virtual crypt model. For instance, in metronomic chemotherapy there is application of a continuous low dose of an agent [64]. The results of metronomic therapy can be modeled by setting the value of chemotherapy “Interval” and “Duration” to be equal, so that there will be no gap in time between doses. Then the intensity and period of therapy to achieve cure (zero “Proportion Mutant Cells”) can be determined by parameter sweeping the values of “Duration” and “Lethality”. Another the kind of therapy, differentiation therapy, aims not to kill mutant proliferating cancer cells with a cytotoxic agent, but rather to induce them to differentiate into more normal cells that will undergo terminal differentiation and therefore not continuing to proliferate. Differentiation therapy has been successfully applied to leukemia cells [100] and has been suggested for solid tumors [101]. Differentiation therapy can be simulated by changing the “Crypt Divide Max”, and then using parameter sweeping with the Behavior Space tool to determine the values of other parameters that maintain homoeostasis of numbers of crypt cell types. An important consideration of any therapy is the existence of heterogeneity of phenotypes among cells in the tumor population [102]. Heterogeneity could be accounted for in the virtual crypt model by simulating different mutant cell phenotypes, and doing parameter sweeping for each phenotype to determine which range of values of “Interval”, “Duration”, and “Lethality” are effective for each different mutant phenotype. Then, a range of parameter values can be chosen that are effective for all, or most, of the different mutant cell phenotypes.

## Conclusion

An agent-based model of homeostatic cell dynamics of human colon crypts has been developed. It was verified as behaving similarly to real crypts, in that it undergoes monoclonal conversion by neutral drift, forms adenomas by mutations located at the top and the bottom of the crypt, and is robust in recovering from cytotoxic perturbation. This model has several novel features. It reproduces the average number of each of the cell types measured in human biopsy specimens, and it reproduces the quasi-stationary stochastic cell dynamics of human crypts as deduced from the measured variation of cell types between different human crypts. Also, it facilitates *in silico* experiments at the graphical user interface.

### Model availability and requirements

Additional file 1 includes the complete model for the virtual crypt, including the “Interface” section, “Information” section (text description), and “Procedures” section (computer scripts). It is licensed under GNU GPL 3. The model runs on the NetLogo application. NetLogo is a multi-agent programmable modeling environment that runs on Windows PC and Mac platforms. It is an open-source application available at http://ccl.northwestern.edu/netlogo. It is authored by Uri Wilensky and developed at The Center for Connected Learning (CCL) and Computer-Based Modeling.

## Abbreviations

CV: Coefficient of variation = standard deviation divided by mean; MIB-1: Antibody to Ki-67; Ki-67: Antigen protein that appears in the G1 part of the cell cycle of proliferating cells; G1: The gap in time of the cell cycle between cell division and the initiation of DNA synthesis.

## Competing interests

The authors declare that they have no competing interests.

## Authors’ contributions

R.B. wrote the code, suggested new features, and carried out some simulations. D.E.A. initiated the project, made measurements of biopsy specimens, provided biological information, did the statistical analysis, carried out most of the simulations, and wrote the manuscript. Together they interpreted the results of the simulations, wrote the Information section of the model, produced the video, and approved of the final article. All authors read and approved the final manuscript.

## Supplementary Material

Additional file 1 **Virtual crypt model.** The model runs on the open-source multi-platform NetLogo application available at http://ccl.northwestern.edu/netlogo/. Click here for file

Additional file 2 **Implementation of the virtual cyrpt (20.5 MB).** Video and audio. QuickTime Player for PC and Mac is available as free download, http://www.apple.com/quicktime/download/. Click here for file

Additional file 3 **Information.** Includes text that describes how to initiate running the model from the Interface. Describes each of the inputs (buttons, sliders, boxes, and outputs (plots, and boxes) of the interface of the model. Includes criteria for selecting default values, and how to conduct parameter sweeps. Click here for file

Additional file 4 **Procedures.** Includes computer script and comments for the model. Click here for file

Additional file 5 **Experimental methods.** Includes source of specimen, image acquisition, measurement by image analysis, and determination of reliability of measurements. Click here for file

Additional file 6 **Cell numbers of the virtual crypt (18.1 MB).** Video and audio. QuickTime Player for PC and Mac is available as free download, http://www.apple.com/quicktime/download/. Click here for file

Additional file 7 **Monoclonal conversion (18.6 MB).** Video and audio. QuickTime Player for PC and Mac is available as free download, http://www.apple.com/quicktime/download/. Click here for file

Additional file 8 **Induction of adenoma (12.5 MB).** Video and audio. QuickTime Player for PC and Mac is available as free download, http://www.apple.com/quicktime/download/. Click here for file

Additional file 9 **Cancer chemotherapy (14.9 MB).** Video and audio. QuickTime Player for PC and Mac is available as free download, http://www.apple.com/quicktime/download/. Click here for file
