# Supplementary material for: A calibrated agent-based computer model of stochastic cell dynamics in normal human colon crypts useful for in silico experiments
Source: Theor Biol Med Model. 2013 Nov 18;10:66. doi: 10.1186/1742-4682-10-66 (PMC3879123; doi:10.1186/1742-4682-10-66)
Supplement: Additional file 3 — Information. Includes text that describes how to initiate running the model from the Interface. Describes each of the inputs (buttons, sliders, boxes, and outputs (plots, and boxes) of the interface of the model. Includes criteria for selecting default values, and how to conduct parameter sweeps. [file 1742-4682-10-66-S3.docx]

**AGENT-BASED COMPUTATIONAL MODEL OF THE COLON CRYPT: DEVELOPMENT OF A VIRTUAL CRYPT FOR IN-SILICO EXPERIMENTS**

January 8, 2013
Coded by Rafael Bravo
Written by Rafael Bravo and David Axelrod
Department of Genetics, Rutgers University, Piscataway, NJ 08854-8082

**INTRODUCTION:**

Colon crypts are invaginations in the large intestine. They are shaped like a test tube with cells arranged along the sides of the test tube. The function of the colon crypts is to produce mucus to lubricate the feces above the crypt in the lumen of the colon, and remove water from the feces. Several different kinds of cells can be recognized, quiescent stem cells at the bottom, proliferating cells (including active stem cells and transient amplifying cells) near the bottom third, and differentiated non-proliferating cells near the top two-thirds of the crypt. Cells produced near the bottom of the crypt move up and are removed at the top of the crypt.

This is an agent-based model in which the crypt has emergent properties resulting from the behavior of a population of individual cells. Cell types are not fixed, rather at each iteration a given cell has a probability of dividing or dying, or dividing and then dying. A cell's probability is determined by its position in a divide gradient and a die gradient. The divide gradient value is greatest at the bottom of the crypt, and the die gradient value is greatest at the top. A cell that divides produces a progeny cell in the same position as the progenitor and a progeny cell that is placed to the right and below the progenitor cell. A cell that dies allows cells below it to move up. These two process result in a flow of cells from the bottom to the top of the crypt, a stochastic variation in the total number of cells per crypt, and in the number of each type of cell.

The goals of this model are (1) to simulate numbers of cell types measured in biopsies of human crypts, and (2) to facilitate in silico experiments. These experiments provide information about the effect of mutations that affect probability of cell proliferation, cell death, orientation of cell division, and response to cytotoxic therapy, among others. Parameter values of normal and mutant cells, and their microenvironment gradients, can be input at the Interface without changing the code. Outputs of stochastic cell dynamics are shown as plots and as digital values in the Interface, and can be exported in spreadsheet format for further analysis.

**PROGRAM OVERVIEW:**

Three views are available. The “Interface” view has a visual representation of the simulated crypt, buttons and sliders for input, and simulation outputs as plots of cell numbers as a function of time. The “Information” view has the description of the program. The “Procedures” view has the commented code.

The Interface view includes a picture of a crypt with three types of cells: quiescent stem cells at the bottom of the crypt in blue, proliferating cells (active stem cells and transient amplifying cells) in the bottom third of the crypt in red, and differentiated cells at the top two thirds of the crypt in blue; mutant cells, if they exist, are yellow. The lamina propria surrounding the crypt cells is brown. The lumen above the crypt is black.

The initial properties of each cell type and cell environment (gradients of extracellular ligands) are specified with buttons and sliders. Outputs of simulations include a picture of different cell types by color and location, and plots as a function of time of the three cell types.

If a cell divides one of the progeny cells move to the lower right, displacing cells previously in that position. When cells reach the right edge of the crypt then they wrap around to the left. If a cell dies, cells below it move up. If a cell reaches the top of the crypt it is removed.

**INITIATION**

“Setup”: initializes the world, the height of the world is determined by "Top", the width by "CellsPerRow", and the size of the initial crypt by "DepthCellsAtStart", these can only be modified before setup.

“Go”: button that initiates the simulation. When clicked, all of the functions are implemented for repeated runs. A series of runs can be stopped by pressing the “Go” button again, which pauses the simulation at the conclusion of the most recent run.

“Defaults”: sets input parameters to their default values as specified in the code. default values and justifications for these values can be found in the Procedures

**APPLIED BY SETUP**

“SetTop”: height of the world in which the crypt exists.

“SetCellsPerRow” : number of cells across crypt. Width of world is number of cells per row plus 2 on each side.

“RowsAtStart” : at beginning of simulation, sets number of cells in each column.

**INPUTS:**

**MUTATES CELL BY ADDING THESE VALUES TO NORMAL CELL PROBABILITIES**

“MutantDivideDiff”: the probability of a mutant cell dividing at a given location in the crypt is changed by adding the value of "MutantDivideDiff" to the value of the cell's own pobdivide determined by its position in the divide gradient. This causes a mutant cell to divide with a different probability than normal cells in the same position.

“MutantDieDiff”: the probability of a mutant cell dying at a given location in the crypt is changed by adding the value of "MutantDieDiff" to the value of the cell's own pobdie determined by its position in the die gradient. This causes a mutant cell to die with a different probability than normal cells in the same position.

**MUTATES ONE OR MORE CELLS**

“MutateOneCell”: button turns one cell from the crypt into a mutant. It appears as a yellow cell, and all of its progeny inherit the mutant trait. Mutant cells are altered by "MutantDivideDiff" and "MutantDieDiff", but are otherwise identical to normal cells.

“MutateDepth”: the number of rows from the top that a single cell will be produced.

"MutateRows": button mutates a "Proportion" of cells in rows between "StartD" and "EndD", where D (Depth) is the number of cell rows from the top of the crypt.

"StartD": start of mutation region for "MutateRows", see above.

"EndD": end of mutation region for "MutateRows", see above.

"Proportion": the proportion of cells between "StartD" and "EndD" that will be mutated when the "MutateRows" button is pressed.

**APPLIES CHEMOTHERAPY**

"ActiveChemo": toggle "On" begins a dose of cytotoxic therapy.

"Interval": the number of ticks from the beginning of one dose to the beginning of the next dose. The time between doses is "Interval" minus "Duration"

"Duration": the number of ticks during which a dose of chemotherapy is applied.

"Lethality": this constant is multiplied by each cell's own probddivide. The product is the probability that the cell will be killed in a given tick of therapy, and removed.

Example (dose = D, no dose = n):
Duration 1, Interval 3: DnnDnnDnnDnnDnnDnnDnnD
Duration 3, Interval 9: DDDnnnnnnDDDnnnnnnDDD

**TOGGLES BETWEEN POLAR AND RANDOM CELL DIVISION ORIENTATION**

"PolarDivision": when toggle is "On", after division one of the progeny cells moves to the right, either 1 or 2 cell positions, and down 1 cell position. This displaces the cell at that location, forcing it to in turn move with the same displacement as the progeny cell. This process continues until there is an empty space for the last displaced cell to occupy. When toggle is "Off", the progeny cell and the cells it displaces move down either diagonally to the left or right 1 cell position, or directly down one cell position.

**RESIZES CRYPT USING CPTDIEMAX**

"CryptSizeAdjust": toggle "On" allows the user to request a steady state size for the crypt, and the program will attempt to meet this size by correcting for the crypt being too large or too small. The program accomplishes this by modifying the "CptDieMax" variable to kill off more or fewer cells per tick, eventually resulting in a properly sized crypt. Toggle "Off" disables this feature.

"AdjustTime": the duration during which the crypt size will be adjusted, counting ticks from setup. The longer this period, the more time "CryptSizeAdjust" will have to reach the requested size, and the more likely a correct crypt size will result.

"CellTarget": target cell number per crypt.

"AdjustStr": determines how quickly the "CptDieMax" variable will be adjusted to compensate for the crypt growing too large or small. A higher value will make the crypt less likely to blow up or extinguish during the "AdjustTime", however it may cause the program to overcompensate for small fluctuations in crypt size.

**CRYPT PRESENTATION**

“ViewProgeny” : toggle "Off" sets cell color based on type, proliferating cells are colored red, with a lighter shade indicating a higher probability of dividing. Differentiated cells, and quiescent stem cells, which have an extremely low probability of dividing, are colored blue, with a lighter shade cooresponding to a higher probability of dying. Toggle "On" assigns each cell a different random color, this color is inherited by the cell's progeny.

“ShowGradients”: toggle "On" displays colored gradients to the left and right of the crypt cells A green divide gradient is displayed on the left and a red die gradient on the right. The quiescent region which is coded as a gradient but whose power is set to 0, making a it a uniform region, is colored dark green, indicating a 0 probability of dividing in this region. Toggle "Off" doesn't display the gradients and allows the program to run faster.

“DiffCellProbabilityThreshold” : cells with a probability of dividing above the threshold value are considered proliferating cells in visuals and plots. Conversely, cells with a probability of dividing below this value are considered differentiated cells or quiescent cells depending on their location.

**CRYPT GRADIENTS**

Gradients: divide and die gradients are described by the following function: p = y^n, where 0<=p=<1, 0<=y=<1, p is the probability that a cell will divide or die, y is related to the range of the gradient, y is raised to the n power. y = 0 at top of the gradient, and y = 1 at the bottom of the gradient. Also, y is scaled so that at the bottom of the gradient y = “Min” and at the top it equals "Max".

The left three inputs affect the divide gradient, the the right three inputs affect the die gradient.

"CptDivMin", "CptDieMin": cooresponds to the gradient value at the minimum end of the gradient. The p = y^n function is scaled so that when y is at it's minimum for the gradient, p = Min.

"CptDivMax", "CptDieMax": cooresponds to the gradient value at the maximum end of the gradient. The p = y^n function is scaled so that when y is at it's maximum for the gradient, p = Max.

"CptDivPwr", "CptDiePwr": cooresponds to the n in the p=y^n function,i.e. the "power" to which y is rasied. This value determines how steeply the gradient approaches it's maximum value.

"QuiesceDepth”: slider that sets the distance from the top of the crypt to the top of the quiescent region. The quiescent region extends from the "QuiesceDepth" to the bottom of the world.

"DieDepthEnd": slider that sets the end of the die gradient. The die gradient extends from the top of the crypt to "DieDepthEnd".

**GRADIENT FEEDBACK STRENGTHS**

"DivideFbk": affects all cells in the crypt above the quiescent region. For a value of 1, at each tick a cell's "ProbDivide" is set to the value at it's height in the divide gradient. For values less than 1, a cell's "ProbDivide" is set to a value determined by both its value at the previous tick and the value of the gradient at the cell's new position in the gradient. This results in a delay in a cell responding to the new gradient value. For a value of 0, the cell is unaffected by the gradient, and retains its previous probdivide.

"DieFbk": affects all cells in the crypt above "DieDepthEnd". similarly to "DivideFbk", a value of 1 sets cell's "ProbDie" to the value of the gradient at the cell's height. For values less than 1, a cell's "ProbDie" is set to a value determined by both its value at the previous tick and the value of the gradient at the cell's new position in the gradient. This results in a delay in a cell responding to the new gradient value.For a value of 0, the cell is unaffected by the gradient, and retains its previous probdie.

"QuiescentFbk": affects all cells in the quiescent region. With a strength of 1, the cells immediately drop to probdivide = 0. With a strength of less than 1, the cell's probdivide will decriment over time, approaching 0. With a strength of 0, the Quiescent region has no effect.

**OUTPUTS:**

**PLOTS OF CELLS AS A FUNCTION OF TIME**

Graphs as a function of time (ticks, iterations) of each of the following:

“Total Cells”: number of all cells in crypt.

“Differentiated Cells”: number of cells which have a probability of dividing greater than "DiffCellProbabilityThreshold" and are located above the quiescent region + "CutOffAboveQuiescentRegion" (this variable is set in the Procedures under setup).

“Proliferating Cells”: number of cells which have a probability of dividing greater than “DiffCellProbabilityThreshold”, but not including quiescent stem cells. This can include cells which are traditionally referred to as active stem cells as well as transient amplifying cells.

“Quiescent Stem Cells”: number of cells which have a probability of dividing less than “DiffCellProbabilityThreshold” and are located below the quiescent region + "CutOffAboveQuiescentRegion".

“Proportion of Mutant Cells” : number of mutant cells divided by total number of cells.

**TIMES TO EVENTS**

"UnboundedSizeTime": if the number of cell rows becomes greater than "SetTop", the crypt is considered unbounded. The "UnboundedSizeTime" reporter displays the number of ticks when this occurs.

"ExtinctionTime": if all of the cells die (removed), the crypt is considered extinct. The "ExtinctionTime" reporter displays the number of ticks when this occurs.

"MonoclonalTime": when the "Viewprogeny" toggle is "On" each cell is assigned a different random color, and this color is inherited by the cell's progeny. The "MonoclonalTime" reporter displays the time period between the time that "Viewprogeny" is toggled "On" and the time that all progeny cells in a crypt have the same color, i.e. that monoclonal conversion has occurred.

"FissionTime": the "FissionTime" reporter displays the period of time between when "PolarDivision" is toggled "On" and the time at which least one column occurs with no cells. A column with no cells that separtes two groups of columns with cells is considered to indicate crypt fission.

**PARAMETER SWEEPING EXPERIMENTS:**

“Behavior space” is accessed under "Tools". This produces numerical output of simulations. Output can be saved in an .csv format file, useful for export to Excel or statistical programs for descriptive statistics and graphs. It is possible to designate parameters to be changed, range of parameters, and intervals. For instance, with two parameters the program will run through the upper and lower limits of each the two parameters, each at designated intervals. The output will include a list of all of the parameter values for the simulation, not just those being swept. For more information see NetLogo User Manual, accessible under "Help".

**CRITERIA FOR SELECTING DEFAULT VALUES:**

The first three can only be changed before activating "Setup". Others can be changed after activating "Setup", but the mouse will need to be clicked outside of any input to activate a new value.

Top (100): convenient value for visualization. If larger, then can have larger number of crypt cells, if too large simulation is slower.

CellsPerRow,RowsAtStart (38): starts the simulation with approximately the number of cells per row and column measured in human crypts.

MutantDivideDiff(0.16): an example value that produces a mutant cell with a greater probability of dividing from others at the same location in the divide gradient.

MutantDieDiff (0.1): an example value that produces a mutant cell with a greater probability of dying from others at the same location in the die gradient.

StartD,EndD (55,60): cells mutated between these rows have a high probability of producing viable mutant progeny rather than dying out.

Proportion (0.2): set so that not all cells between start depth (StartD) and end depth (EndD) are mutated.

MutateHeight (59): Starting at this height, compared to other heights, a mutant cell is more likely to produce viable progeny rather than die out.

Interval, Duration, Lethality (24,3,2): given a lethality of 2, these interval and duration values give a minimum collateral damage with a short time to cure.

CellTarget (2428): average total number of cells measured in human crypts

AdjustTime,AdjustStr (1000,0.1): give "CryptSizeAdjust" a sufficient number of ticks and sufficient strength to achieve the target number of cells.

DiffCellProbabilityThreshold (0.02): gives the approximate numbers of differentiated, proliferating, and quiescent cells measured in human crypts.

CptDivMin,CptDivMax,CptDivPwr (0,0.5,11.5): this combination of values causes cell division behavior that simulates the numbers and proportion of each cell type as measured in human crypts.

CptDieMin,CptDieMax,CptDiePwr (0,0.2,14): this combination of values causes cell death behavior near the top of the crypt to simulate cell migration out of the crypt.

QuiesceDepth (60): simulates the measured total number of cells per crypt, also simulates the approximately measured number and proportion of quiescent cells.

DieDepthEnd (100): causes the die gradient to cover the entire crypt depth with SetTop as its default value.

DivdeFbk,DieFbk,QuiescentFbk (0.5,0.5,0.1): these give intermediate rates of delayed response of cells to their gradient position.

**ACKNOWLEDGEMENTS:**

This crypt model was produced with the NetLogo v.4.3.3 application. NetLogo is a multi-agent programmable modeling environment. It is authored by Uri Wilensky and developed at The Center for Connected Learning (CCL) and Computer-Based Modeling. It is an open-source application available at <http://ccl.northwestern.edu/netlogo/>.
