# Supplementary material for: A calibrated agent-based computer model of stochastic cell dynamics in normal human colon crypts useful for in silico experiments
Source: Theor Biol Med Model. 2013 Nov 18;10:66. doi: 10.1186/1742-4682-10-66 (PMC3879123; doi:10.1186/1742-4682-10-66)
Supplement: Additional file 4 — Procedures. Includes computer script and comments for the model. [file 1742-4682-10-66-S4.docx]

; Rafael Bravo, VirtualCryptModel020413G

; Nov. 26 2012, Jan. 8, 2013,

; Jan. 31, 2013 (Comment out duration = 8x interval)

;

;This crypt model was produced with the NetLogo v.4.3.3 application.

;NetLogo is a multi-agent programmable modeling environment.

;It is authored by Uri Wilensky and developed at

;The Center for Connected Learning (CCL) and Computer-Based Modeling.

;It is an open-source application available at http://ccl.northwestern.edu/netlogo/.

;

; This program is free software: you can redistribute it and/or modify

; it under the terms of the GNU General Public License as published by

; the Free Software Foundation, version 3 of the License.

;

; This program is distributed in the hope that it will be useful,

; but WITHOUT ANY WARRANTY; without even the implied warranty of

; MERCHANTABILITY or FITNESS FOR A PARTICULAR PURPOSE. See the

; GNU General Public License for more details.

;

; You can receive a copy of the GNU General Public License

; at <http://www.gnu.org/licenses/>.

globals[ ;variables that are accessed by mutliple methods (globally) are named here

keepgoing ;set as false when either the crypt is reduced to a single layer of cells or the crypt grows over the top of the world.

colorprogeny ;toggles whether color displays probdivide or cell ancestry.

MonoclonalTime; used to display how many ticks it takes for monoclonal conversion to occur

ExtinctionTime; displays the tick at which the crypt collapses

UnboundedSizeTime; displays the tick at which the crypt produces polyps (overflow)

CellsInLumen; displays the number of cells specifically in the lumen, above the crypt.

FissionTime; time it takes the crypt to split into multiple crypts after random cell div. is turned on

;Protrusion; distance from the lumenal surface that cells protrude (usually zero)

highest; depth of the crypt

chemoct ; used to keep track of the chemotherapy interval.

colored ; binary, keeps track of whether progeny are colored or not.s

top; size of world, altered by setup

CellsPerRow ; number of cells per row in crypt, altered by setup

CutOffAboveQuiescentRegion ; the cutoff between what is called a quiescent and a differentiated cell, since they are both defined as having a probability of dividing lower than DiffCellProbabilityThreshold

; is done spacially, by drawing a line "CutOffAboveQuiescentRegion" cell rows above the quiescent gradient, and all cells above this line are counted as differentiated, while cells below are counted as quiescent

; this indicator may miscount cells if the proliferating region is too small, and may need to be modified for the counts and plots below to print the desired cell groups.

CountSCq ; globals display respective cell types, useful for easier reporting in behavior space.

CountProlif

CountDiff

CountMutant

MONOCLONALCOMPLETE; temporary variable

] ;called by the cells every cycle, generates a random float from 0 to 1 similar to a "dice roll" to determine whether cells in the crypt divide and/or die.

breed [cells cell]

cells-own [; variables that the cells themselves posess are named here

probdivide ;float from 0 to 1, a cell will divide if rand is less than this value. imposed by applygradientchancedivide and can be influenced if cell is mutated.

probdividegoal

probdie ;float from 0 to 1, a cell will die if rand is less than this value. imposed by applygradientchancedie and can be influenced if cell is mutated.

probdiegoal

changespeeddiv; these are used for delayed feedback, stores the feedback of the current gradient

changespeeddie

mutant ;Boolean value, if true cell is considdered mutant and colored blue, MutantDivideDiff, MutantDieDiff, and IgnoreQuiesce only apply to mutated cells.

]

to setup ; properly sizes the world, and populates it with the specified number of cells OBS ONLY

let qdtemp QuiesceDepth; temporary variables store QuiesceDepth and RowsAtStart, which are sliders that have top as their maximum, so that the settings of these variables are not lost when top is set to 0 by clearall

let rastemp RowsAtStart

let ddetemp DieDepthEnd

clear-all

set CutOffAboveQuiescentRegion 5

set top SetTop

set RowsAtStart rastemp

set QuiesceDepth qdtemp

set DieDepthEnd ddetemp

set-default-shape cells "circle"

set CellsPerRow SetCellsPerRow

resize-world -4 cellsPerRow + 3 0 top + 2 ; world is sized so that it fits around the crypt with four spaces of buffer to the left and right. two spaces of buffer are added to the top of the crypt.

; y coordinates move up from 0 at bottom of the world to top+2 at the top.

set-patch-size 400 / top ; resizes world so that it will fit in the window space alotted, (Does not work in all cases, if crypt is too wide, world will overlap buttons)

let x 0

let y top

while [y > top - RowsAtStart][

set x 0

while [x < cellsPerRow][;this section of code populates the crypt, placing a cell at every location in the crypt from the bottom to RowsAtStart, going row by row.

makecell(.5)(.5)(x)(y) ;makecell creates cells at target location with starting values set.

set x x + 1]

set y y - 1]

set keepgoing true

set chemoct -1

set colored false

;globals are set to their starting values.

ask cells [

set probdivide probdividegoal

set probdie probdiegoal]

; temporary: for behaviorspace!!!!

;set interval duration * 8

set MONOCLONALCOMPLETE false

end

to go ; iterates the simulation OBS ONLY

; these variables are set to help with counting cells

set CountSCq count cells with [ycor < top - quiescedepth + CutOffAboveQuiescentRegion and probdivide < DiffCellProbabilityThreshold and Mutant = false]; variables defined for use with behavior space

set CountProlif count cells with [probdivide >= DiffCellProbabilityThreshold and Mutant = false]

set CountDiff count cells with [probdivide < DiffCellProbabilityThreshold and ycor >= top - quiescedepth + CutOffAboveQuiescentRegion and Mutant = false]

set CountMutant count cells with [Mutant = true]

; two reasons why the simulation would need to stop, either the crypt overflows or the crypt dies, the rest of the simulation assumes neither of these occured.

if count cells = 0 and keepgoing = true[set keepgoing false set ExtinctionTime ticks print (word "the crypt died at tick " ExtinctionTime)]; if there is less than one full row of cells, cell death and division stops, and the crypt is considdered dead.

if count cells with [ycor = top + 2]> 0 and keepgoing = true [ set keepgoing false set UnboundedSizeTime ticks print (word "the crypt overflowed at tick" UnboundedSizeTime) ]

if keepgoing = true[; keepgoing is set to false if the crypt dies or overflows, prevents simulation from continuing at that point.

; sets up protrusion (how far crypt should stick out into the lumen) and highest (approx number of rows of cells)

set highest round(count cells / cellsperrow); determines the approximate top of the crypt, estimated by the average height of the cell columns.

;ifelse highest > maxcryptdepth [set protrusion highest - maxcryptdepth][set protrusion 0]; if the average height of the cell columns is above the maximum crypt depth, the crypt height is set at maxcryptdepth

ask patches[set pcolor black]; sets all patches as black so the crypt can be redrawn.

if showgradients = false [ask patches[

if pycor <= top [

set pcolor 132]; patches that are considered in the crypt are colored light brown

]]; patches that are considdered outside the crypt are left black

; Sets up and applies gradient functions

; exponential gradient functions take arguments start height, end height, start value, end value

; and power. this scales the function y = x^(power) from zero to 1 so that the

; range is applied from start height to end height and so that the y value is proprtionally

; gradient values scaled to range between startvalue and endvalue.

applygradientprobdivide(top)(top - highest)(cptdivmin)(cptdivmax)(cptdivpwr)(DivideFbk) ;feedbackdiv set to quiescefeed so that both could be modified in parallel in behavior space.

applygradientprobdie(top - DieDepthEnd)(top)(cptdiemin)(cptdiemax)(cptdiepwr)(DieFbk) ;applies gradient that sets probdie of cells at every iteration

;applygradientprobdivide(top - protrusion + 1 )(top - protrusion + LumDivSiz + 1)(lumdivmin)(lumdivmax)(lumdivpwr)(feedbackdiv) ; 2d pair of gradients apply to the lumen

;applygradientprobdie(top - protrusion + 1 )(top - protrusion + LumDieSiz + 1)(lumdiemin)(lumdiemax)(lumdiepwr)(feedbackdie)

applygradientprobdivide(0)(top - quiescedepth)(0)(0)(0)(QuiescentFbk) ;quiescent gradient applies to cell division.

ask cells[set probdivide probdivide + (probdividegoal - probdivide) * changespeeddiv

set probdie probdie + (probdiegoal - probdie) * changespeeddie ; gradients are applied to cells, cells are only affected by the last probdivide and probdie gradients, in order as in the code

; all cells are given a chance to divide and die at every iteration.

; cell division and death occurs here

let rand random-float 1 ; generates a random floating point number from 0 to 1 for every comparison called rand

if rand < probdivide [

hatch-cells 1

makespace(self) ; if rand is less than the probdivide, the cell reproduces

]]

ask cells [; after cell proliferation is taken care of, all cells have a probability of dying.

let rand random-float 1

if rand < probdie [ ; if rand is less than probdie, the cell dies

die

]]

; sets up and applies chemotherapy

if activechemo = true and chemoct = -1 [

set chemoct 0] ; initiates chemotherapy

if activechemo = false and chemoct > -1 [set chemoct -1] ; stops chemotherapy if activechemo is set to false

if chemoct >= 0 [

if chemoct < duration[

ask cells[

let rand random-float 1

if rand / lethality < probdivide[

die]

]

]

ifelse chemoct = interval[set chemoct 0]

[set chemoct chemoct + 1]

]

shiftdown; keeps cell columns without gaps by forcing cells to move up if there is a space above them.

; sets up cell coloring under normal conditions, if viewprogeny is true cells are not colored.

if viewprogeny = false ; colors cells the standard colors, differentiated and quiescent cells are colored based on the height at which they first transitioned from actively dividing to differentiated

; actively dividing cells are colored based on their probablility of dividing.

[set colored false

set MonoclonalTime 0

ask cells [if probdivide <= DiffCellProbabilityThreshold [set color probdie / CptDieMax * 5 + 94]

if probdivide > DiffCellProbabilityThreshold [set color probdivide / CptDivMax * 9 + 11]

]]

ask cells with [mutant = true][set color 45] ; changes color of mutant cells to yellow so that they are easily visible. yellow color is retained even if colorprogeny is true

; sets up FissionTime reporter

ifelse PolarDivision[

set FissionTime 0][

let emptycolumn false

let x 0

repeat CellsPerRow [

if count cells with [xcor = x] = 0[

set emptycolumn true]

set x x + 1] ; iterates through all cell columns, seeing if any of them are empty

if emptycolumn = false [

set FissionTime FissionTime + 1]; if no empty column exists, fission time will continue to be incremented

]

; if viewprogeny is set to true, this section will handle cell coloring, as well as check whether monoclonal conversion has yet occured.

if viewprogeny = true and colored = false[

ask cells [

set color random-float 140 ; when this function is called and colorprogeny is set to true, all cells are given a random color

]

set colored true]

if viewprogeny = true and colored = true[

let increment false

let checkcolor 0

ask one-of cells[

set checkcolor color]

ask cells [

if checkcolor != color[set increment true]]

ifelse increment = true[set MonoclonalTime MonoclonalTime + 1][

set MONOCLONALCOMPLETE true]]; if all cells have the same color, then monoclonal conversion has occured.

; misc.

;ifelse count cells - cellsperrow * Maxcryptdepth > 0 [

; set CellsInLumen cellsperrow * protrusion]

; [set CellsInLumen 0]; reports the number of cells that are protruding into the lumen

do-plot ; plots cell populations at the end of each iteration.

if AdjustTime > ticks and cryptsizeadjust = true [

adjustcptdiemax] ; applies cryptsizeadjust if it is being used.

]

tick; view is updated at every tick

end

to makecell[setprobdivide setprobdie x y];places a cell with specified parameters at the specified x y coordinates, called by setup, OBS ONLY

create-cells 1 [

setxy x y

set probdivide setprobdivide; setprobdivide and setprobdie are specified at the method call.

set probdie setprobdie ; if cell falls within gradient limits, these values will be overwritten by gradient

set mutant false

set color 9.9

]

end

to mutaterows;turns proportion cells between rows startH and endH mutant, startH and endH counting from bottom of crypt

if startD > endD [let temp startD

set startD endD

set endD temp]

ask cells with [top - ycor >= startD and top - ycor <= endD][

let rand random-float 1

if rand < proportion[

set mutant true]]

end

to makespace[celltomove]; forces cells out of the way to make room for a newly generated cell

let currx [xcor] of celltomove

let curry [ycor] of celltomove

let nextx 0

ifelse polardivision [

set nextx random 2 + 1 + currx]

[set nextx random 3 - 1 + currx]; depending on whether or not divdirrand is on, cells will either move preferentially to the right, or move down with no right-left preference.

let nexty curry - 1

if nextx < 0 [

set nextx CellsperRow + nextx]

if nextx >= CellsperRow [

set nextx nextx - CellsperRow]

let nextcell one-of cells with [xcor = nextx and ycor = nexty]

ask celltomove[

setxy nextx nexty

while [count turtles-at 0 1 = 0 and ycor != top][setxy xcor ycor + 1]]

if nextcell != nobody [

makespace(nextcell)]

end

to shiftdown ;forces all cells up in columns so there are no gaps in the crypt.

let y top - 1

while [y >= 0][; goes from the top of the world to the bottom.

ask cells with [ycor = y and count cells-at 0 1 = 0][; gets all cells with an empty space above them in a particular row,

let movedown 0

while[count cells-at 0 (movedown + 1) = 0 and y + movedown <= top - 1][

set movedown movedown + 1] ; finds the highest space above the cell that does not contain a cell.

setxy xcor ycor + movedown ] ; moves the cell to this location

set y y - 1]

end

to do-plot ; parameters used in conjunction with plots, called by Go function at the end of each iteration, does not affect simulation itself.

set-current-plot "Total Cells"

set-current-plot-pen "cellspen"

plot count cells ; total cells plot displays total number of cells.

set-current-plot "Quiescent Stem Cells"

set-current-plot-pen "CountQuiescentpen"

plot count cells with [ycor < top - quiescedepth + CutOffAboveQuiescentRegion and probdivide < DiffCellProbabilityThreshold] ; Quiescent Stem Cell plot diplays number of quiescent cells. (blue cells)

set-current-plot "Proliferating Cells"

set-current-plot-pen "CountProliferatingPen"

plot count cells with [probdivide >= DiffCellProbabilityThreshold];Displays number of cells that are not quiecent and have probdivide grater than DiffCellProbabilityThreshold. (red cells)

set-current-plot "Differentiated Cells"

set-current-plot-pen "CountDifPen"

plot count cells with [probdivide < DiffCellProbabilityThreshold and ycor >= top - quiescedepth + CutOffAboveQuiescentRegion] ; Displays number of cells that have probdivide less than DiffCellProbabilityThreshold. (blue cells)

set-current-plot "Proportion Mutant Cells"

set-current-plot-pen "PropMuts"

if count cells > 0 [plot count cells with [mutant = true]/ count cells] ; Proportion Mutant cells diplays number of mutant cells divided by total number of cells.

end

; The following code creates a gradient effect over the area, startval and endval must be between 0 and 1, startpoint and endpoint must be between the

; beginning and end of the model. note, the variable that the gradient affects cannot be stated as a parameter for the function, so the

; function had to be duplicated to affect probdivide and probdie.

to applygradientprobdivide [starty endy startval endval power feedback] ; gradient that applies to probdivide of all cells between startval and endval.

if starty != endy [

if power < 0[ ; the function used here is x^n, where x is from 0 to 1. the value of x ranges between 0 and 1 regardless of the value of n,

let temp endy

set endy starty

set starty temp

set temp endval

set endval startval

set startval temp] ; this bit of code reverses variables so that negative powers cause the graph's inflection to bow outward from 0 to 1.

let range starty - endy ; graph originally goes from 0 to 1, but is scaled by range and set to go from starty to endy.

let shift startval - endval ; similarly, in x^n from 0 to 1, the value of x goes from 0 to 1, but is scaled by shift and set to go from startval to endval

let y starty

let counter 0

while[counter <= abs(range)][

ask cells with[ycor = y][; all cells in row y are altered by the following function

ifelse shift > 0

[set probdividegoal (- abs((ycor - starty) / range)^ abs(power)) * abs(shift) + startval

if mutant = true[set probdividegoal probdividegoal + mutantdividediff]] ; to apply negative gradient, scaled graph x^n is inverted

[set probdividegoal abs((ycor - starty) / range)^ abs(power) * abs(shift) + startval; positive scaled graph x^n is applied here

if mutant = true[set probdividegoal probdividegoal + mutantdividediff]] ; to apply negative gradient, scaled graph x^n is inverted

set changespeeddiv feedback ; modifies cell feedback value

]

if showgradients = true [ask patches with [pycor = y and pxcor < cellsperrow / 2][; this function modifies the patch color along the gradients.

ifelse shift > 0

[set pcolor 61 + 7 * (- abs((pycor - starty) / range)^ abs(power)) * abs(shift) + startval] ; to apply negative gradient, scaled graph x^n is inverted

[set pcolor 61 + 7 * abs((pycor - starty) / range)^ abs(power) * abs(shift) + startval]]] ;pcolor is affected rather than probdivide and probdie.

ifelse range < 0[set y y + 1][set y y - 1]

set counter counter + 1

]]

end

to applygradientprobdie [starty endy startval endval power feedback] ; a duplicate of applygradientprobdivide, only it affects probdie.

if starty != endy [

if power < 0[

let temp endy

set endy starty

set starty temp

set temp endval

set endval startval

set startval temp]

let range starty - endy

let shift startval - endval

let y starty

let counter 0

while[counter <= abs(range)][

ask cells with[ycor = y][

ifelse shift > 0

[set probdiegoal (- abs((ycor - starty) / range)^ abs(power)) * abs(shift) + startval

if mutant = true[set probdiegoal probdiegoal + mutantdiediff]]

[set probdiegoal abs((ycor - starty) / range)^ abs(power) * abs(shift) + startval

if mutant = true[set probdiegoal probdiegoal + mutantdiediff]]

set changespeeddie feedback ; modifies cell feedback value

]

if showgradients = true [ask patches with [pycor = y and pxcor >= cellsperrow / 2][

ifelse shift > 0

[set pcolor 12 + 7 * (- abs((pycor - starty) / range)^ abs(power)) * abs(shift) + startval] ; to apply negative gradient, scaled graph x^n is inverted

[set pcolor 12 + 7 * abs((pycor - starty) / range)^ abs(power) * abs(shift) + startval]]]

ifelse range < 0[set y y + 1][set y y - 1]

set counter counter + 1

]]

end

to defaults ; sets defaults for all variables modifyable in main interface, see information for justifications for values.

set Settop 100

set RowsAtStart 61; gets crypt into a steady state position as quickly as possible.

set setcellsperrow 38; simulates approximate number of cells to produce proper crypt diameter if wrapped into a cylinder

set quiescedepth 60; produces the proper number of cell rows

set CptDivMin 0; cells will not divide towards the top of the crypt

set CptDivMax .5; gives cells a high probability of dividing near the bottom of the crypt, so that feedback has greater effect

set CptDivPwr 11.5; keeps cell division happening only near the bottom of the crypt.

set CptDieMin 0; cells near the bottom of the crypt have almost zero probability of dividing, with cell removal only happening near the top

set CptDieMax .2; this kills off cells at the proper rate to keep a steady crypt with the right proportions of cells.

set CptDiePwr 14; keeps cell death only happening towards the extremem top of the crypt.

set DieDepthEnd 100; die gradient speads over the entire area of the crypt.

set DiffCellProbabilityThreshold .02; this value only changes the classification of cells in the crypt, and was adjusted to get proper cell proportions.

set mutantdividediff .16; makes mutant cells divide more quickly, one of the hallmarks of mutation leading to cancer.

set mutantdiediff .1; makes mutant cells more likely to die, also typically occurs with early mutant cells (less stability than normal cells.)

set MutateDepth 59

set showgradients false; increases running speed.

set Lethality 2; adjusted to this value to be more effective at killing off cells, since the crypt is more robust.

set activechemo false

set interval 24

set duration 3

set startD 55; ensures mutant cells begin in the quiescent region

set PolarDivision true

set endD 60

set proportion .2

set cryptsizeadjust false; activate if modifying other variables to keep crypt at proper size

set AdjustTime 1000

set CellTarget 2428; approx. number of cells in actual crypt, as observed experimentally.

set AdjustStr .1

set DivideFbk .5; values set to produce cell proportions most similar to those observed.

set DieFbk .5

set QuiescentFbk .1

end

to mutateonecell ; randomly sets a cell in the given row to mutate.

ifelse count cells with [ycor = top - mutatedepth and mutant = false] > 0[

ask one-of cells with [ycor = top - mutatedepth and mutant = false] [set mutant true]][

print "no elligible cells in the chosen row"]

end

to adjustCptDieMax; method is called by CryptSizeAdjust to attempt to set the size of the crypt to the requested value

set CptDieMax CptDieMax + (count turtles - Celltarget)/ Celltarget * (AdjustTime - ticks) / (AdjustTime) * AdjustStr

if CptDieMax < 0 [set CptDieMax 0]

if CptDieMax > 1 [set CptDieMax 1]

end

to togglecolor ; gives all cells a distinct color that is inherited by progeny so monoclonal conversion can be observed

ifelse colorprogeny = false[

set colorprogeny true

][set colorprogeny false] ; if colorprogeny was true, colorprogeny is set to false, and cells will be colored based on probDivide.

end
