# Supplementary material for: A calibrated agent-based computer model of stochastic cell dynamics in normal human colon crypts useful for in silico experiments
Source: Theor Biol Med Model. 2013 Nov 18;10:66. doi: 10.1186/1742-4682-10-66 (PMC3879123; doi:10.1186/1742-4682-10-66)
Supplement: Additional file 5 — Experimental methods. Includes source of specimen, image acquisition, measurement by image analysis, and determination of reliability of measurements. [file 1742-4682-10-66-S5.docx]

Additional file 5: Measurements of cell numbers in human biopsy specimen.docx

*Specimen:* A slide was obtained from Dr. Steven Schiff, Cancer Institute of New Jersey. The slide contained sections from a biopsy of the sigmoid colon of a normal patient. The slide was stained with antibody MIB-1 to the G1 proliferation antigen Ki-67, and counter stained with hematoxylin. Use of human tissue was approved by the Rutgers Institutional Review Board with exception for anonymous specimens.

*Image acquisition:* Color images were captured with Magnifire software 2.1C (Optonics Goleta, CA, www. Optronics.com), using an Optronics Camera, Model S60800 (Monochrome CCD, RGB filters, 6.7 x 6.7 microns pixels, 1300 x 1300 pixels) attached to a Nikon Labophot bright field microscope with a 20X, 0.4 NA objective. Each captured image contained one crypt.

*Measurements of cell numbers:* There were a total of 49 crypts available whose image included a cross-section in which cells could be seen from the bottom, along the middle on both sides, and across the lumen at the top. The number of cells appearing in the two-dimensional image of a cross section of each of 49 crypts were counted and used to calculate the number of cells in a three-dimensional crypt. Images of nuclei were selected with the Point Selection tool of ImageJ v 1.43 software (Wayne Rasband, http://rsb.info.nih.gov/ij). Four measurements were made for each crypt: w (the number of cells from the midpoint of the base to lumen half way to the adjacent crypt), y (the number of cells around the half-circular bottom of the crypt), z (the number of cells on one side of the crypt that are stained with MIB-1 antibody), and usb (the number of cells at the bottom of the crypt that are not stained with MIB-1 antibody). From these measurements, the number of cells in each crypt was determined by assuming that the cells in the crypt were on the surface of a cylinder with the bottom end capped by a hemisphere (http://mathworld.wolfram.com/SphericalCap.html). Cells stained positively for the Ki-67 proliferation antigen were considered to be proliferating cells [83]. Unstained cells at the bottom of the crypt were considered to be quiescent stem cells, and unstained cells near the top of the crypt were considered to be differentiated cells.

*Measurement reliability:* The reliability of measurements was determined by repeatedly measuring only one crypt 49 times. The coefficients of variation of the measurements were the following: w, 3.6%; y, 5.6%, z, 5.6%, usb, 3.5%. And the coefficients of variation of the calculated number of cells per crypt were the following: SCq 7.7%; Prolif, 8.4%; Diff 10.7%; Total 7.2% Since the experimental error in repeated measurements, and calculated cell number from these measurements, was much less than the variation determined between different crypts, it is likely that the variation between crypts is not due just to experimental error but represents real differences between adjacent crypts in the colon of a single patient.

*Statistical analysis.* JMP v.7 (SAS Institute, Cary, NC) was used to perform statistical analysis and plot data.
